# Activity-dependent regulation of the BAX/BCL-2 pathway protects cortical neurons from apoptotic death during early development

**DOI:** 10.1007/s00018-023-04824-6

**Published:** 2023-06-03

**Authors:** Jonas Schroer, Davide Warm, Federico De Rosa, Heiko J. Luhmann, Anne Sinning

**Affiliations:** Institute of Physiology, University Medical Center of the Johannes Gutenberg University, Duesbergweg 6, 55128 Mainz, Germany

**Keywords:** Programmed cell death, Somatosensory cortex, Postnatal development, BCL-2 family, Executor caspases, Neuronal firing

## Abstract

During early brain development, homeostatic removal of cortical neurons is crucial and requires multiple control mechanisms. We investigated in the cerebral cortex of mice whether the BAX/BCL-2 pathway, an important regulator of apoptosis, is part of this machinery and how electrical activity might serve as a set point of regulation. Activity is known to be a pro-survival factor; however, how this effect is translated into enhanced survival chances on a neuronal level is not fully understood. In this study, we show that caspase activity is highest at the neonatal stage, while developmental cell death peaks at the end of the first postnatal week. During the first postnatal week, upregulation of BAX is accompanied by downregulation of BCL-2 protein, resulting in a high BAX/BCL-2 ratio when neuronal death rates are high. In cultured neurons, pharmacological blockade of activity leads to an acute upregulation of *Bax*, while elevated activity results in a lasting increase of BCL-2 expression. Spontaneously active neurons not only exhibit lower *Bax* levels than inactive neurons but also show almost exclusively *BCL-2* expression. Disinhibition of network activity prevents the death of neurons overexpressing activated CASP3. This neuroprotective effect is not the result of reduced caspase activity but is associated with a downregulation of the BAX/BCL-2 ratio. Notably, increasing neuronal activity has a similar, non-additive effect as the blockade of BAX. Conclusively, high electrical activity modulates BAX/BCL-2 expression and leads to higher tolerance to CASP3 activity, increases survival, and presumably promotes non-apoptotic CASP3 functions in developing neurons.

**Supplementary Information:**

The online version contains supplementary material available at 10.1007/s00018-023-04824-6.

## Introduction

The brain initially contains an excess of neurons, and the size of the neuron population is only later adjusted by apoptotic cell death. This process is fundamental for the proper maturation of functional neuronal networks and requires strict regulation via different pathways [1–3]. Key players in the apoptotic process are members of the B-cell lymphoma 2 (BCL-2) family [4, 5]. This protein family has up to four BCL-2 homology (BH) domains and is distinguished by its family members’ pro- or anti-apoptotic functions. Upon stimulation, the BCL-2-associated X protein  (BAX) and BCL-2 homologous antagonist/killer (BAK), members of the pro-apoptotic group, oligomerize in the outer mitochondrial membrane and thereby form pores that lead to the permeabilization of the outer mitochondrial membrane. Thereupon, cytochrome *c* is released and triggers the apoptotic pathway. BCL-2 is the direct antagonist of BAX and inhibits oligomerization directly interacting with BAX or by preventing BH3-only proteins, a subfamily of the pro-apoptotic group, from activating BAX [6, 7]. The importance of the BCL-2 protein family for the proper development of the nervous system was demonstrated in different transgenic mouse studies [8–13]. Here, anti-apoptotic family members, like myeloid cell leukemia 1 (MCL-1) or B-cell lymphoma 2 like 1 (BCL-xL), are crucial for the survival of immature, newborn [9, 13, 14], and early postmitotic neurons [15]. They play not only important roles during the first apoptotic wave [16], but also in the migration process of cortical neurons [13, 15]. BCL-2 and consequently its overexpression protect neurons from developmental apoptosis, but is also involved in the survival and maintenance of mature neurons throughout life [5, 10, 17]. The structural similarities of this protein family allow for high interactivity between members, which prompted the hypothesis that the balance between pro- and anti-apoptotic factors, in particular BAX and BCL-2, determines whether a cell survives or dies [4, 7].

A second wave of apoptosis occurs in the rodent cortex at the end of the first postnatal week and reduces neuronal populations in a cell-type- and region-specific manner [18–21]. Notably, at the same time as this wave of apoptosis abates, oscillatory patterns of activity emerge [22]. Synchronized network activity controls the fate of developing cortical neurons and networks [23]. Blocking or desynchronizing neuronal activity increases the probability of cell death while increasing synchronized activity has a positive, if not decisive, effect on cell survival [19, 21, 24–26].

On the single-cell level, neurons that display distinct firing patterns, such as burst activity associated with high intracellular Ca^2+^ fluxes, are less likely to die [25, 27]. Electrical activity and Ca^2+^-dependent mechanisms as regulators of gene expression, e.g., of immediate early genes (IEGs), were noted as early as the 1980s [28]. Here, the increase in intracellular Ca^2+^ initiates a multitude of signaling cascades [29, 30], some of which are known to be beneficial for neuronal survival, repair, and maintenance in the peripheral and central nervous system [31–33]. In all these models, neuronal loss is prevented in mice lacking BAX [12, 19, 31, 34]. Important regulators of neuronal survival, such as the AKT serine–threonine kinase 1 (AKT) and its counterpart phosphatase and tensin homolog (PTEN), are also impacted in expression and function by neuronal activity [19, 35, 36]. Although the above mentioned studies show that electrical activity is important for neuronal survival and affects gene expression, it is not yet clear how this pro-survival effect of activity controls the fate of individual neurons in developing networks.

In this study, we investigated which apoptotic pathways are regulated during early cortex development, how neuronal activity affects this regulation, and thus how electrical activity is translated into neuron survival. We first compared the developmental course of cell death, and overall caspase activity, as well as the key players in the mitochondrial apoptosis pathway BAX and BCL-2 in the cortex during postnatal mouse development. The results show that despite the pronounced activation of caspase 3 (CASP3) during the execution of developmental death [37, 38], overall caspase activity declines throughout postnatal development. In contrast, the relative BAX/BCL-2 ratio is highest when postnatal cell death is at its peak. Only in active neurons this ratio is shifted toward the pro-survival side. To test for the activity-dependent regulation of the neuronal BAX/BCL-2 ratio, we used primary cortical cultures of wild-type and genetically modified mice in which active neurons can be labeled. The high caspase activity in the cortex of newborn pups suggests that neurons can sustain high caspase activity without directly executing the cell death program. To assess whether in this scenario neuronal activity is sufficient to increase the individual tolerance for CASP3 activity in neurons, we overexpressed the active form of CASP3 using an adeno-associated virus (AAV) vector approach and analyzed the effect of pharmacological manipulation of activity. Overall, the results of these experiments suggest that the presence of electrical activity reduces the relative BAX/BCL-2 expression in immature neurons, whereby tolerance for CASP3 activity is selectively raised in active neurons with a higher likelihood of survival during early cortical development.

## Materials and methods

### Animals

All experiments were conducted in accordance with the National and European (2010/63/EU) laws for the use of animals in research and were approved by the local ethical committee (Landesuntersuchungsamt Rheinland-Pfalz, 23 177-07/G 14-1-080 and G 20-1-006). Offspring from timed-pregnant C57BL/6NRj wild-type mice (Janvier Labs) were used for most experiments. For a subset of experiments, either Arc^CreERT/+^/R26^AI14/+^ or GAD67-GFP knock-in mice [39] were used. Double transgenic Arc^Cre/+^/R26^AI14/+^ mice were achieved by crossing heterozygous B6.Cg-Tg(Arc-Cre/ERT2)MRhn/CdnyJ mice [40] with homozygous B6.Cg-Gt(ROSA)26Sortm14(CAG-tdTomato)Hze/J [41]. For the preparation of protein lysates for Western blot analysis, animals younger than postnatal day (P)7 were killed by decapitation, and older mice by cervical dislocation. For TUNEL analysis of cortical samples, mice older than P3 were transcardially perfused with PBS supplemented with heparin followed by 4% formaldehyde (FA) fixation. For P1 mice, animals were killed by decapitation. Brains were removed and fixed overnight in 4% FA at 4 °C.

### Primary neuronal cultures

In vitro experiments were performed in primary cortical neurons cultured from newborn (P0) C57BL6J/NRj, double heterozygous Arc^CreERT/+^/R26^AI14/+^, or heterozygous GAD67-GFP mice. After decapitation, brains were transferred to ice-cold Ca^2+^- and Mg^2+^-free HBSS (Gibco, Invitrogen) supplemented with penicillin and streptomycin (50 units/mL), sodium pyruvate (11 mg/mL), glucose (0.1%), and HEPES (10 mM). Cortical cells were dissociated via trypsin incubation for 20 min at 37 °C and an additional 5 min with DNase. After washing with HBSS, trypsinization was blocked with minimal essential medium (MEM, Gibco) supplemented with 10% horse serum and 0.6% glucose. Next, the cells were mechanically dissociated via repetitive pipetting through fire-polished glass pipettes with declining diameters and filtration through a 40-µm cell strainer (Greiner). Following cell counting, cells were seeded on poly-l-ornithine-coated glass coverslips, or cell culture imaging chambers (IBIDI) (density: 2500 cells/mm^2^). After 25 min, medium was exchanged with a medium consisting of Neurobasal medium (Gibco) supplemented with 2% B27 (Gibco) and 1 mM l-glutamine. Cells were cultivated at 37 °C and 5% CO_2_ in humidified air. After 2 days, 5 µM AraC was added to the medium to inhibit glial cell proliferation. Once a week one-third of the medium was removed and replaced with half the initial volume of BrainPhys Neuronal medium plus SM1 supplement (StemCell Technology).

### Viral transduction of primary cortical neurons

Primary neurons were transduced after 1-3 days in vitro (DIV) with recombinant AAV (rAAV1/2; appr. 1–4 × 10^4^ copies/cell) either carrying pAAV-Ef1a-mCherry-IRES-Cre (Neuro-Cre) or pAAV-hDlx-mCherry-IRES-Cre (GABA-Cre) and at DIV7 with pAAV-flex-taCasp3-TEVp (FLEx-Casp3). pAAV-Ef1a-mCherry-IRES-Cre was a gift from Karl Deisseroth (Addgene plasmid # 55632; http://n2t.net/addgene:55632; RRID:Addgene_55632); pAAV-flex-taCasp3-TEVp was a gift from Nirao Shah and Jim Wells (Addgene plasmid # 45580; http://n2t.net/addgene:45580; RRID:Addgene_45580). For the generation of the GABA-Cre plasmid, the Ef1a promoter in the Neuro-Cre plasmid was replaced with a hDlx promoter (originally derived from Addgene plasmid # 83896 (http://n2t.net/addgene:83896; RRID:Addgene_83896; a kind gift from Gordon Fishell). This delayed co-transduction protocol led to a stable mCherry-Cre expression without interfering in the establishment of the network and facilitated the rapid aCASP3 overexpression thereafter. For a subset of experiments, neurons were transduced at DIV 1 with recombinant AAV carrying pAAV-hSyn-H2B:GFP for nuclear labeling (modified as described previously [42]). Viral production was done according to During et al. [43]. In brief, HEK293 cells were co-transfected with plasmids carrying the rep and capsid sequence of AAV serotypes 1 and 2, a helper plasmid, and the above-described plasmid carrying the genes of interest flanked by inverted terminal repeat (ITR) sequences. After 48 h, rAAV was harvested, extracted, and purified via heparin columns (HiTrap® Heparin columns, Sigma-Aldrich). Viral titers were determined by quantitative real-time PCR.

### Tamoxifen application in vitro

(*Z*)-4-Hydroxytamoxifen (4-OH-Tam) (Sigma-Aldrich; Cat# H7904) was dissolved at a stock concentration of 10 mM in ethanol and stored at −20 °C. For experiments, 4-OH-Tam was further diluted in medium to a final concentration of 1 µM and applied at different DIV as indicated. For subsequent longitudinal imaging, live or fixed cultured neurons were imaged at different developmental time points with a 20 × objective (UPlanFL 20x/0.5 na, Olympus) on an epifluorescence microscope (IX81, Olympus) connected to a CCD camera (XM10, Olympus) using the cellSens Software (Olympus). For the quantification of cell fate, bright-field and fluorescence signals (nuclear H2B-GFP and somatic Arc-Cre tdTomato) were followed over time with the help of gridded imaging chambers (IBIDI). The AAV-based overexpression of a nuclear tag H2B-GFP (see details above) allowed the unbiased identification of apoptotic neurons with evident nuclear condensation as described previously [27, 38]. Arc-Cre/tdTomato expression allowed the identification of neurons that were active in the effective period upon one-time tamoxifen application. In a subset of experiments, multiplexed FISH (see below) was performed 24 h after 4-OH-Tam application.

### Caspase-Glo assay

Caspase activity was measured by the luminescent Caspase-Glo® 3/7 Assay (Promega). This homogeneous, luminescent assay provides a luminogenic caspase 3/7 substrate, which contains the tetrapeptide sequence DEVD, in a reagent optimized for caspase activity, luciferase activity, and cell lysis. The protocol was performed according to the manufacturer’s guidelines. In short, for in vitro applications, equal amounts of Caspase-Glo® 3/7 reagent and PBS were added to the cultured neurons upon removal of the medium. Buffer and cells were mixed and incubated for 30–45 min. For whole cortex measurements, cortices were isolated and lysed in 10 µl PBS per mg of cortex. Equal volumes of cell lysate and Caspase-Glo® 3/7 reagent were mixed and incubated for 90 min. Luminescence was measured with a Tecan infinite M1000 plate reader (Tecan Group) and normalized to controls for statistical analysis.

### Multiplexed fluorescence in situ hybridization (FISH)

FISH analysis was performed with the ViewRNA ISH cell assay kit (QVC0001, Invitrogen) for in vitro samples and the ViewRNA ISH tissue kit (QVT0700, Invitrogen) for brain slices. Probes were designed using Invitrogen’s ViewRNA ISH assay probe sets tool. In the multiplex approach, the following probes were used to label RNA of *Bax* (Type 4), *BCL-2* (Type 6), and *cFos* (Type 1). The protocols were performed according to the manufacturer’s guidelines with slight adaptations. In brief, fixed cells or 30-µm coronal brain slices were stored in PBS with DEPC to ensure RNA integrity until hybridization was performed. After permeabilization, the samples were incubated with probe sets. Following different washing steps, the signal was amplified. In the final step, the amplified signal was labeled with different fluorochromes. Here, *Bax* was labeled with FITC signal, *cFos* with Cy3, and *BCL-2* with Cy5 fluorochrome. Afterward, cells were mounted with Immunoselect Antifading Mounting Medium with DAPI (DIANOVA) and dried overnight at room temperature (RT) in the dark.

Images from fixed cultures were taken with a 40 × objective (UPlanFLN 40x/0.75na, Olympus) with an epifluorescence microscope (IX81, Olympus) connected to a CCD camera (XM10, Olympus) using the cellSens Software (Olympus). Images were subsequently analyzed in Fiji [44]. Depending on the signal density of the different probes, either number of puncta or mean signal intensity per cell was analyzed. In detail, for analysis of *Bax* and *BCL-2* probe signals, the total number of puncta/cell were counted. Due to high signal density and intensity of the *cFos* signal in activated neurons upon Gbz application, somatic *cFos* signal intensities were compared for cultured neurons after pharmacological treatments in Fig. 3.

Images of brain slices were taken with a 60 × water objective (CFI Plan Apo VC 60XWI, 1.2 NA) at a spinning disk confocal microscope (Visitron Systems) with a CSU-W1 spinning disk (25 and 50 µm pinholes, Yokogawa) connected to a Prime BSI sCMOS camera (2048 × 2048 pixels, 6.5 µm pixel size, Photometrics) using the VisiView software (Visitron Systems). Subsequent analysis was performed in Fiji. To achieve single-cell resolution, 6-µm-thick optical slices were analyzed. Optical slices were generated by maximal projection of 6 subsequent images in the *z*-direction with 1 µm imaging depth. mRNA expression was analyzed only in the cortical plate (P1) or layers II/III and IV (P7). Therefore, layers were marked and cell numbers were automatically counted based on the DAPI signal with particle analysis in Fiji. FISH puncta were then normalized to the number of cells for comparable results. For single-cell analysis of RNA particles, only isolated, non-overlapping cells were included in the analysis. Puncta located in the somatic area with a diameter 50% larger than the nucleus were assigned to individual cells, and for all probes, the number of puncta/cell was quantified.

If *Bax* and *BCL-2* signals were analyzed for individual cells, the *individual Bax/BCL-2 ratio* was calculated as the relative number of *Bax* and *BCl-2* punta/cell or displayed in a group-wise analysis. For comparative analysis of Bax/BCL-2 ratios per field of view (FOV) or across active and inactive populations, the *average Bax/BCL-2 ratio* was calculated as the mean *Bax* and *BCL-2* expression per FOV or across analyzed cell populations.

### Protein samples and Western blot analysis

For cortical samples, intact cortices of mice at different postnatal ages were snap-frozen and stored at −20 °C. The tissue was disrupted physically and further lysed in lysis buffer (Thermo Scientific). For cell lysates, cell medium was replaced with lysis buffer and lysed for 30 min at 4 °C. Afterward, lysates were centrifuged at 16,000*g* for 10 min at 4 °C, and the supernatant was used for further analysis. Protein concentrations were measured via BSA assay and adjusted to 2.5 µg/µl for cortex and 1 µg/µl for cell lysates in loading buffer with 50 mM dithiothreitol. 20–30 µg proteins were resolved by SDS-PAGE and transferred onto a PVDF membrane. Membranes were then blocked with 4% milk in Tris-buffered saline solution (TBS) with 0.1% (v/v) Tween 20 (Sigma-Aldrich) and incubated with primary antibodies, also in TBS-T, overnight at 4 °C. Membranes were washed with TBS-T before incubation with HRP-linked secondary antibodies for 1 h at RT. HRP signal was detected using ECL solution (Invitrogen), and imaging was done with the ChemiDoc System (Bio-Rad). The following primary and secondary antibodies were used: primary: mouse monoclonal BAX (MA5-14003, Invitrogen, 1:250), mouse monoclonal BCL-2 (SC-7382, Santa Cruz Biotechnology, 1:250), rabbit polyclonal BCL-xL (#2762, Cell Signaling Technology, 1:500), polyclonal CASP3 (#9662, Cell Signaling Technology, 1:500), and rabbit monoclonal beta-ACTIN (ab115777, Abcam, 1:2000) and secondary: goat anti-mouse IgG (H + L) secondary antibody, HRP (31430, ThermoFisher, 1:10000), and goat anti-rabbit IgG (H + L) secondary antibody, HRP (31460, ThermoFisher, 1:10000).

### Cell death labeling (TUNEL)

For TdT-mediated dUTP-biotin nick end labeling (TUNEL) analysis, FA-fixed brains were washed with PBS (0.1 M, pH 7.5), and cortical hemispheres were separated. After cryoprotection in 30% sucrose in phosphate buffer overnight at 4 °C, 20-µm sagittal slices were cut on a freezing microtome (Leica CM 1325; Leica Mikrosysteme). Slices were collected in PBS 0.1 M, mounted on SuperfrostTM Plus Adhesion microscope slides left to air dry at RT, and subsequently stained in a humid chamber. Unspecific signals were blocked with 7% normal donkey serum and 0.3% triton diluted in PBS for 2 h at RT or with 0.1% tri-sodium citrate 0.8% triton diluted in PBS for 1 h at RT for TUNEL assay. The reaction mixture (In Situ Cell Death Detection Kit Fluorescein, Roche Diagnostics) was applied for 2 h at 37 °C. Thereafter, tissue sections were washed three times with PBS and incubated with DAPI. Specimens were mounted with Fluoromount (Sigma-Aldrich). Images were acquired with a 10 × objective (UPLFLN10X2PH, Olympus) with an epifluorescence microscope (IX81, Olympus) connected to a CCD camera (XM10, Olympus) using the cellSens software (Olympus). Quantitative analysis of TUNEL-positive neurons was performed independently of cortical areas, i.e., the total number of TUNEL-positive neurons was determined throughout the cortex in sagittal brain slices 1–2 mm lateral to the midline. Among others, the cortical section analyzed included S1, M1, and V1 regions, for which representative images are also shown. Images were subsequently analyzed in Fiji.

### Immunocytochemistry

For immunocytochemistry, cells were pre-fixed in 2% formaldehyde for 5 min and then fixed in 4% formaldehyde in phosphate buffer (ROTI® Histofix 4%, Carl Roth) for 15 min. After washing with PBS, cells were stored at 4 °C until further use.

To reduce background fluorescence, cells were quenched with 0.1 M NH_4_Cl for 10 min at RT. Unspecific binding of antibodies was blocked and cells were permeabilized with normal donkey serum (017-000-121, Jackson ImmunoResearch)/0.3% (v/v) triton (Triton® X-100, Sigma-Aldrich) in PBS 0.01 M for 2 h at RT. Afterward, cells were incubated overnight at 4 °C in PBS 0.01 M/2% bovine serum albumin (001-000-161, Jackson ImmunoResearch)/0.05% sodium azide (S002, Sigma-Aldrich)/0.1% triton with the following primary antibody: rabbit monoclonal anti-NeuN (ab177487, Abcam,1:200). For fluorescence labeling, the following fluorophore-conjugated secondary antibody was used: Alexa Fluor647-conjugated AffiniPure anti-rabbit IgG (H + L) (Jackson ImmunoResearch 1:200).

Images from fixed neurons were taken with a 10 × objective (UPLFLN10X2PH, Olympus) with an epifluorescence microscope (IX81, Olympus) connected to a CCD camera (XM10, Olympus) using the cellSens software (Olympus). Images were subsequently analyzed in Fiji.

### Real-time PCR

RNA was extracted and purified using the RNeasy Micro kit (50) (Qiagen). Gene expression analysis was performed using iTaq Universal SYBR Green Supermix (Bio-Rad) and the StepOnePlus real-time PCR system (Applied Bioscience). Primers for *Bax*, *BCL-2*, *cFos,* and *Actin* were designed using the Pick primers function provided by the National Library of Medicine and obtained from ThermoFisher Scientific. The housekeeping gene *Actin* was used as a reference, and relative gene expression was calculated using the ΔΔct method.

### Multi electrode array (MEA) recordings and calcium imaging

Extracellular electrical recordings were performed as described previously [45]. In short, cells were cultured on MEAs containing 120 planar extracellular titanium nitrite electrodes with four internal references (120MEA100/30iR-Ti-gr, Multi Channel Systems). MEAs had an electrode diameter of 30 μm and an interelectrode spacing of 100 µm. Signals from 120 recording electrodes were recorded for 5 min with MC_Rack software in an MEA2100 system (Multi Channel Systems) at a sampling rate of 50 kHz and high-pass filtered at 200 Hz. Spikes were detected using a negative threshold-based detector set to 5.5 × the SD of the noise level (MC_Rack, Multi Channel Systems). Spike datasets from all electrodes were then imported into Plexon software for analysis of single units using a spike sorting algorithm. Spike waveforms were sorted with the automatic sorting method ‘Valley Seeking Scan’ with the following parameters: Parzen multiplier range of 0.5–1.5 and steps of 0.2. Only units, that fired ≥ 2 times within the recording period, were counted as active neurons. Simultaneous Ca^2+^ imaging was performed with an upright microscope (OlympusBX61WI) equipped with a Hamamatsu Orca R2 C10600 CCD camera. 1 µM Oregon Green 488 BAPTA 1, AM (O6708, ThermoFisher) was used to record Ca^2+^ signals. Network activity was extracted by the MEA recordings, and Ca^2+^ signals were used to calculate the participation of single neurons in this network activity.

### Statistics

For all experiments, the total number of biological replicates is indicated as *n*. Independent biological replicates are noted as *N*. Technical replicates were included as averages in the statistical analysis. Values are given as mean values ± SEM. All statistical tests were performed using GraphPad Prism 9 (GraphPad). Comparisons between multiple groups were made either with a one-way ANOVA followed by Tukey’s multiple comparison test for post hoc analysis or by Kruskal–Wallis test followed by Dunn’s multiple comparisons test if one group did not pass the Shapiro–Wilk test. A two-way ANOVA followed by Šidák’s multiple comparison test was applied, when applicable. Comparisons between two groups were made with Student’s unpaired and paired t-test or nonparametric Mann–Whitney test when applicable. Differences in proportions were calculated using the Chi-square test. Significance was considered at *p* values < 0.05.

## Results

### Caspase activity continuously decreases, while BAX/BCL-2 ratio peaks with developmental apoptosis in the postnatal mouse cortex

**Fig. 1:**
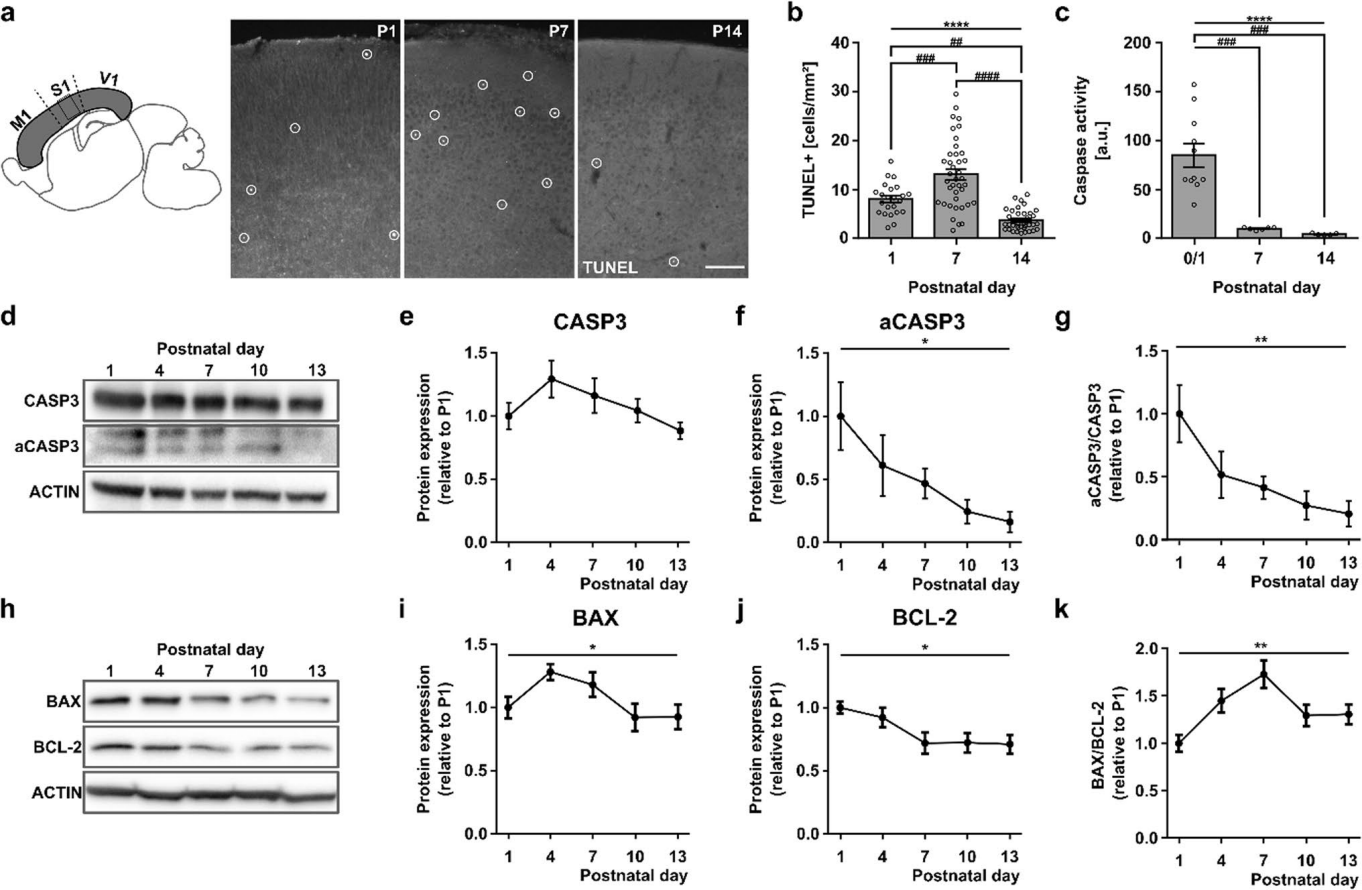
Developmental regulation of key players in the mitochondrial apoptosis pathway, BAX and BCL-2, but not of caspase activity, coincides with postnatal cell death in the mouse cerebral cortex.** a** Representative photographs of the somatosensory cortical region of TUNEL stained sagittal sections at P1, P7, and P14 (scale bar = 100 µm). **b** Quantification of cortical cell death across the entire cortex (gray region in **a**) in sagittal slices 1–2 mm lateral of midline during the first two postnatal weeks (*n* = 23, 39, 37 fields of view from *N* = 5 mice each; one-way ANOVA, ****p < 0.0001, post hoc Tukey’s multiple comparisons test ^###^ P1 vs P7 *p* < 0.001, P1 vs P14 ^##^*p* < 0.01, P7 vs P14 ^####^*p* < 0.0001). **c** Caspase 3/7 activity measured by Caspase-Glo assay is highest at P0/1 and sharply declines during the first two postnatal weeks (*n* = 10; 6; 5 cortical lysates from *N* = 10; 6; 5 mice; one-way ANOVA, ****p < 0.0001; post hoc Tukey’s multiple comparisons test: P0/1 vs P7 ^###^*p* < 0.001, P0/1 vs P14 ^###^*p* < 0.001, P7 vs P14 *p* > 0.05). **d** Representative Western blot against full-length and cleaved CASP3. **e**–**g** Quantification of Western blots of full-length (**e**) and cleaved (**f**) CASP3 (*n* = 8 cortices from *N* = 8 mice, one-way ANOVA, *p* > 0.05; *p < 0.05) expression levels and the relative aCASP3/CASP3 ratio (**g**) from P1 to P13 (*n* = 8 cortices from *N* = 8 mice, one-way ANOVA **p < 0.01). **h** Representative Western blot against BAX and BCL-2. **i**–**k** Quantification of Western blots of BAX (**i**) and BCL-2 expression levels (**j**) (*n* = 10 cortices from *N* = 10 mice, one-way ANOVA, *p < 0.05; *p < 0.05) and the relative BAX/BCL-2 ratio (**k**) from P1 to P13 (*n* = 10 cortices from *N* = 10 mice, one-way ANOVA **p < 0.01)

To investigate whether the postnatal peak in apoptosis in the murine cortex can be explained by caspase activity regulation, we characterized cell death and caspase activity at different postnatal time points. In line with previous reports [18, 19, 46], the occurrence of TUNEL-positive cells/mm^2^ within the cortex was higher at P7 compared with P1 and P14 (Fig. 1a, b, Suppl. Figure 1, P1: 8.01 ± 0.68 vs P7: 13.12 ± 1.12 vs P14: 3.68 ± 0.35, one-way ANOVA, *p* < 0.0001). Next, we performed the luminescent caspase-Glo assay on cortical lysates at different postnatal time points (P0/1, P7, and P14). Caspase 3/7 activity in cortical lysates was highest at P0/1 and significantly decreased at the end of the first and second week, respectively (Fig. 1c, P0/1: 84.6 × 10^3^ ± 10.3 × 10^3^ vs P7 9.88 × 10^3^ ± 1.30 × 10^3^ vs P14: 4.29 × 10^3^ ± 0.95 × 10^3^ a.u., one-way ANOVA, *p* < 0.0001). Western blot analysis of CASP3 expression in cortical lysates taken from P1 to P13 (Fig. 1d) confirmed that while expression of the full-length CASP3 did not significantly change (Fig. 1e, P1: 1.00 ± 0.10 vs P4: 1.29 ± 0.14 vs P7: 1.16 ± 0.14 vs P10: 1.04 ± 0.09 vs P13: 0.89 ± 0.07, one-way ANOVA, *p* > 0.05), CASP3 activity, i.e., expression of cleaved CASP3 fragments, continuously decreased during the first two postnatal weeks (Fig. 1f, P1: 1.00 ± 0.27 vs P4: 0.62 ± 0.24 vs P7: 0.47 ± 0.12 vs P10: 0.25 ± 0.09 vs P13: 0.17 ± 0.08, one-way ANOVA, *p < 0.05). As a result, the relative aCASP3/CASP3 expression decreased significantly (Fig. 1g, P1: 1.00 ± 0.23 vs P4: 0.52 ± 0.18 vs P7: 0.42 ± 0.09 vs P10: 0.28 ± 0.11 vs P22: 0.22 ± 0.10, one-way ANOVA, *p* < 0.01). Hence, the overall caspase 3/7 activity sharply declines during the first two postnatal weeks, while cortical neuronal death peaks at the end of the first postnatal week.

The pro-apoptotic factor BAX is known to play an essential role in programmed cell death [8] and is developmentally downregulated later in life when apoptosis rates are low [47]. To determine the expression levels of BAX and its pro-survival counterpart BCL-2 on different days during the first and second postnatal weeks, we first performed Western blot analysis on whole cortices of wild-type mice (Fig. 1h). The results showed that BAX, compared to P1, was stronger expressed at the end of the first postnatal week and decreased only in the second postnatal week (Fig. 1i, P1 1.00 ± 0.09 vs P4: 1.28 ± 0.06 vs P7: 1.18 ± 0.10 vs P10: 0.92 ± 0.11 vs P13: 0.93 ± 0.10, one-way ANOVA, *p* < 0.05). In contrast, BCL-2 expression decreased with development until it reached a plateau toward the end of the first postnatal week (Fig. 1j, P1 1.00 ± 0.05 vs P4: 0.92 ± 0.08 vs P7: 0.72 ± 0.08 vs P10: 0.72 ± 0.08 vs P13: 0.71 ± 0.08, one-way ANOVA, *p* < 0.05). As a result, the BAX/BCL-2 ratio peaked at P7 (Fig. 1k, P1 1.00 ± 0.09 vs P4 1.45 ± 0.12 vs P7: 1.73 ± 0.15 vs P10: 1.30 ± 0.11 vs P13 1.31 ± 0.10, one-way ANOVA, *p* < 0.01). The expression of BCL-xL, another pro-survival factor of the BCL-2 family, was also analyzed by Western blot. During the first postnatal week, BCL-xL expression decreased sharply and was very low at P7 (Suppl. Figure 2). This expression profile supports previous data, which demonstrated an important role of BCL-xL for survival in early postmitotic neurons only directly after birth [15].

**Fig. 2:**
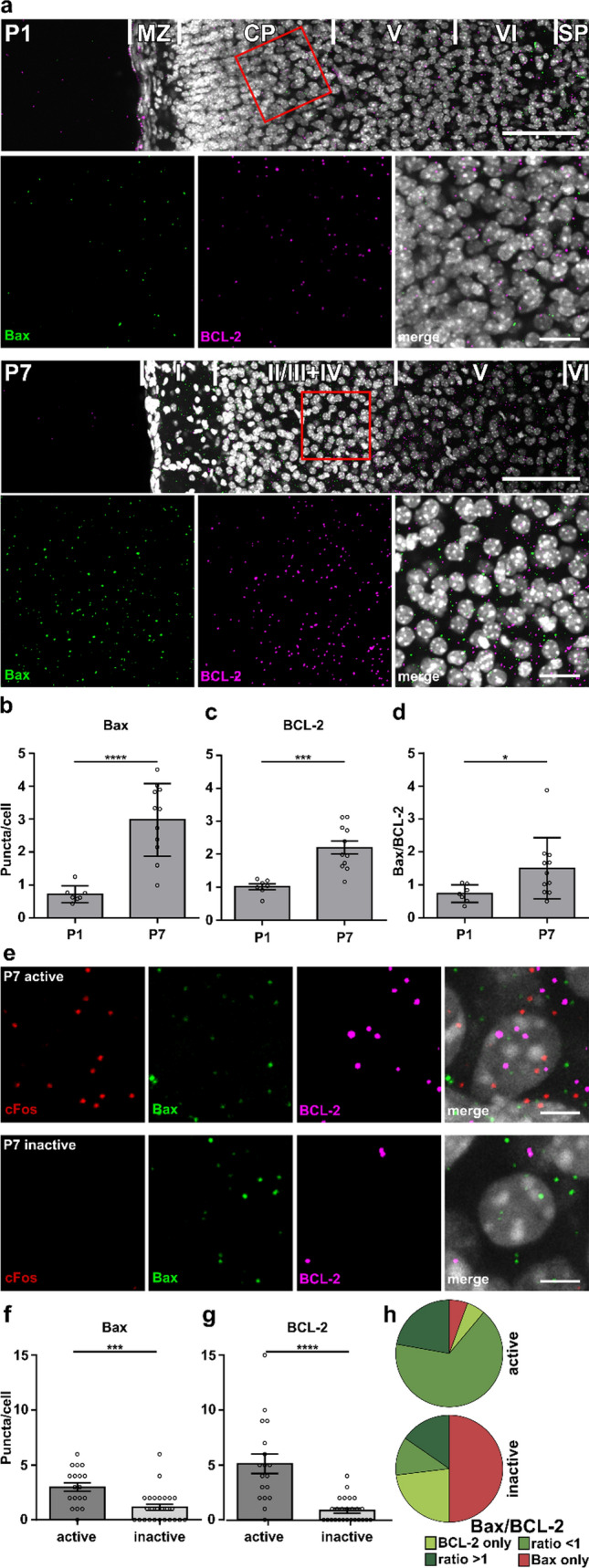
*Bax/BCL-2* ratio is higher at P7, but active neurons have a lower ratio than inactive neighbors.** a** Representative images of FISH against *Bax* and *BCL-2* in the cortex at P1 and P7 (overview, scale bar = 100 µm, inset, scale bar 25 µm, *MZ* marginal zone; *CP *cortical plate; *SP* subplate). **b**–**d** Quantification of *Bax* (**b**) and *BCL-2* (**c**) puncta in the CP at P1 and layer II/III and IV at P7 normalized to the number of cells in the field of view and the resulting *Bax/BCL-2* ratio (**d**) for each age (*n* = 7, 11 fields of view from *N* = 4 brain slices, in **b** unpaired Student’s *t* test, ****p < 0.0001, in **c** unpaired Student’s *t* test, ***p < 0.001, in **d**, unpaired Student’s *t* test, *p < 0.05). **e** Representative images of FISH against *cFos*, *Bax,* and *BCL-2* of single cells in layers II/III and IV in the cortex of P7 animals. Individual cells were selected using DAPI and a round area with a diameter 50% larger than the nucleus was analyzed (scale bar = 5 µm). **f**, **g** Quantification of *Bax* (**f**) and *BCL-2* (**g**) puncta per cell. Cells were considered as active if they had at least 5 *cFos* puncta and as inactive if they had a maximum of 1 *cFos* puncta (*n* = 18, 26 from *N* = 5  brain slices, in **f** Mann–Whitney test, ***p < 0.001, in **g** Mann–Whitney test, ****p < 0.0001). **h** Group-wise comparison of *Bax/BCL-2* ratio (Chi-square, *** *p* < 0.001)

To investigate the developmental regulation of *Bax* and *BCL-2* on the transcriptional level, we performed multiplexed FISH against *Bax* and *BCL-2* in brain slices at P1 and P7. Since in the developing rodent cortex, the vast majority of cells are neurons [48], we compared expression levels of *Bax* and *BCL-2* normalized to the cell number in the cortical plate at P1 and layers II/III and IV at P7 (Fig. 2a). In good agreement with our Western blot results, expression levels of *Bax* increased significantly between P1 and P7 (population average: Fig. 2b, P1 0.72 ± 0.10 vs P7: 2.98 ± 0.33 puncta/cell, unpaired Student’s t test, *p* < 0.0001, single neuron average: Suppl. Figure 3a, b). For *BCL-2,* average mRNA expression levels (i.e., number of *BCL-2* normalized to the cell number per FOV) also increased from P1 to P7 (population average: Fig. 2c, P1 1.02 ± 0.09 vs P7: 2.20 ± 0.20 puncta/cell, unpaired Student’s *t* test, *p* < 0.001). However, if individual neurons were analyzed, there was no significant change (Suppl. Figure 3 a, c). As a result, the strong increase in absolute *Bax* expression from P1 to P7 led to a significantly higher *Bax/BCl-2* ratio at P7 (population average: Fig. 2d, P1 0.74 ± 0.10 vs P7: 1.51 ± 0.28, unpaired Student’s *t* test, *p* < 0.05, single neuron average: Suppl. Figure 3d).

Taken together, these results demonstrate that caspase activity progressively decreases with postnatal age, while the expression of BAX and BCL-2 on protein and mRNA level is regulated such that the pro-apoptotic BAX/BCL-2 ratio peaks at a time point associated with high levels of apoptosis during postnatal development in the mouse cortex.

### At P7, active neurons show a lower Bax/BCL-2 ratio as compared to inactive neurons

Single cell analysis of multiplexed FISH signals for *Bax*, *BCL-2*, and *cFos* probes at P7 revealed that most layer II/III and IV neurons are *cFos* negative or show very few *cFos* puncta (average *cFos* puncta/cell: 1.67 ± 0.06) and thus considered as electrically inactive neurons (Fig. 2e). Consequently, the average *Bax/BCL-2* ratio of *cFos*-negative neurons, calculated as the average *Bax* and *BCL-2* expression per cell, was comparable to that described for layers II/III and IV on the population level (P7: single neuron average 1.36 vs population average 1.51). But interestingly, the few active neurons, which showed high *cFos* expression with at least 5 *cFos* puncta/cell, displayed high absolute levels of *Bax* (Fig. 2f, active: 3.00 ± 0.40 vs inactive: 1.15 ± 0.28 puncta/cell, Mann–Whitney test, *p* < 0.001) with even higher absolute *BCL-2* levels (Fig. 2g, active: 5.11 ± 0.89 vs inactive: 0.85 ± 0.21 puncta/cell, Mann–Whitney test, *p* < 0.0001). As a result, the average *Bax/BCL-2* ratio of active neurons is decreased to an anti-apoptotic value of 0.59 on average. In more detail, half of the inactive cell population (13/26) did not show *BCL-2* expression, which made the calculation of the individual *Bax/BCL-2* ratio for single neurons not possible. We therefore, grouped cells into four categories (*BCL-2 only*, *more BCL-2: Bax/BCL-2* ratio < 1, *more Bax: Bax/BCL-2* ratio > 1, *Bax only*) and compared their distributions between active neurons (high *cFos*, with at least 5 *cFos* puncta/cell) and inactive neurons (no or maximum 1 *cFos* puncta/cell) (Fig. 2h). The proportions differed significantly with most of the active cells (12/18) in the *more BCL-2* group and only a few (4/18) in the *more Bax* group. In contrast, most inactive cells (13/26) expressed only *Bax*, and only in a minority of cells (3/26) was the *Bax/BCL-2* ratio smaller than 1 (Chi-square, *p* < 0.001).

In summary, at P7, active neurons display a lower *Bax/BCL-2* ratio than inactive neurons. This shift in the ratio is due to an increase in *BCL-2* expression in active neurons compared to inactive neurons and suggests that *Bax* and *BCl-2* are not only regulated developmentally but also in an activity-dependent manner.

### Acute and long-term activity-dependent regulations of the BAX/BCL-2 pathway

**Fig. 3:**
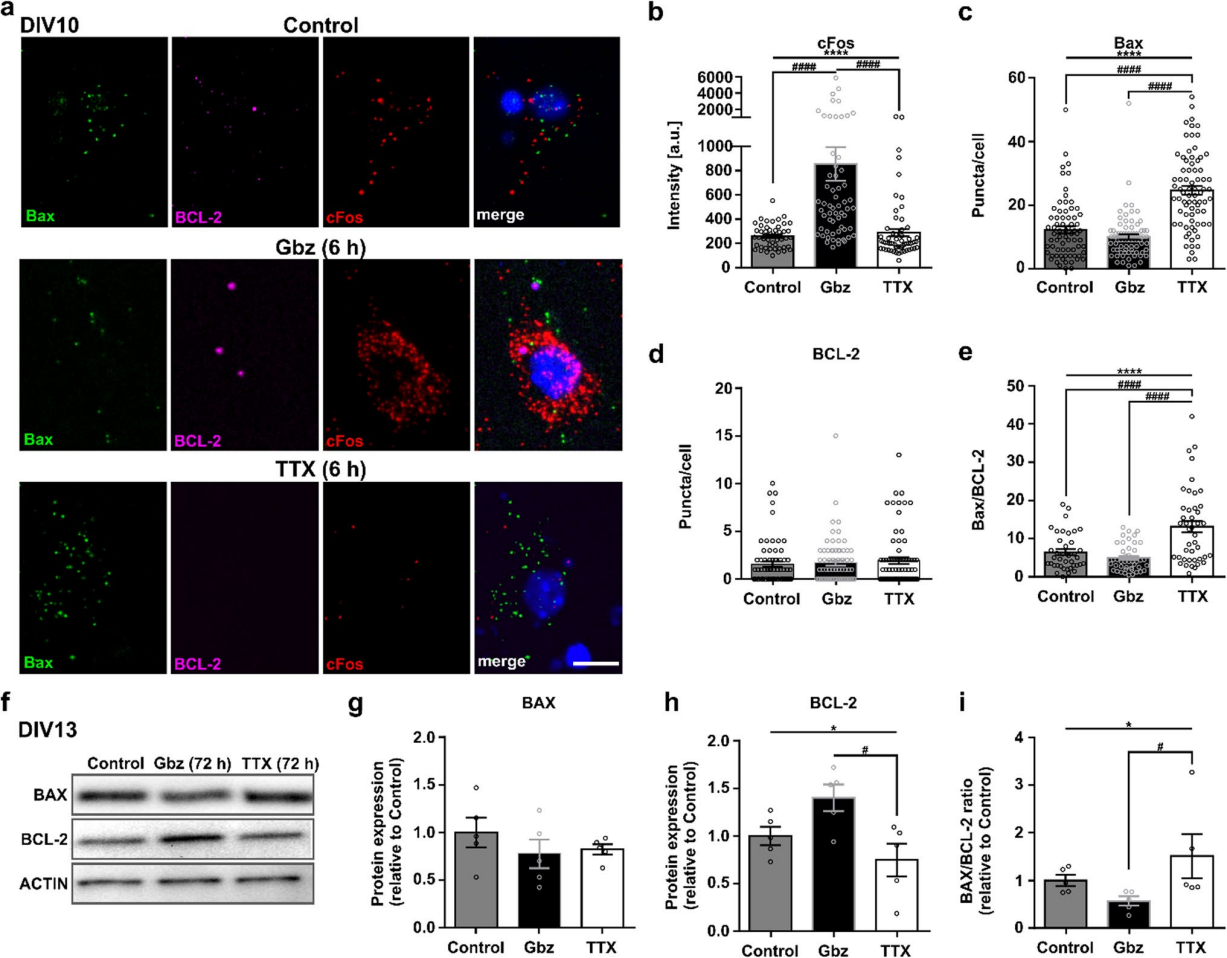
Acute and long-term modulation of electrical activity alters the BAX/BCL-2 ratio. **a** Representative images of multiplexed FISH against *Bax, BCL-2,* and *cFos* in primary cortical neurons from newborn (P0) mice under control conditions, Gbz, or TTX treatment for 6 h at DIV10 (scale bar = 10 µm). **b** Increasing neuronal network activity significantly increases neuronal *cFos* expression analyzed as mean signal intensity per cell (*n* = 60, 60, 48 cells from *N* = 3 cultures each, one-way ANOVA, ****p < 0.0001, post hoc Tukey’s multiple comparisons test: control vs Gbz ^####^*p* < 0.0001, control vs TTX *p* > 0.05, Gbz vs TTX ^####^*p* < 0.0001). **c** Blocking activity with TTX strongly increases the number of *Bax* puncta per cell, i.e., *Bax* expression, whereas increasing electrical activity with Gbz has no significant effect (*n* = 71, 74, 75 cells from *N* = 3 cultures each, one-way ANOVA, ****p < 0.0001, post hoc Tukey’s multiple comparisons test: control vs Gbz *p* > 0.05, control vs TTX ^####^*p* < 0.0001, Gbz vs TTX ^####^*p* < 0.0001). **d** *BCL-2* expression in cortical neurons, i.e., number of *BCL-2* puncta per cell, is not significantly altered by pharmacological manipulation of electrical activity (*n* = 71; 74; 75 cells from *N* = 3 cultures each, one-way ANOVA, *p* > 0.05). **e** The relative ratio of *Bax/BCL-2* puncta/cell is significantly increased when neuronal activity is blocked by TTX application (*n* = 39, 44, 43 cells from *N* = 3 cultures each, one-way ANOVA ****p < 0.0001, post hoc Tukey’s multiple comparisons test: control vs Gbz *p* > 0.05, control vs TTX ^####^*p* < 0.0001, Gbz vs TTX ^####^*p* < 0.0001). **f** Representative Western blots against BAX and BCL-2 in cell cultures treated at DIV10 for 72 h with either Gbz or TTX. **g**–**i** Quantification of Western blots against Bax (**g**) and BCL-2 (**h**) (*n* = 5 from *N* = 4 culture preparations, one-way ANOVA, *p* > 0.05, *p < 0.05; post hoc Tukey’s multiple comparisons test: control vs Gbz *p* > 0.05, control vs TTX > 0.05, Gbz vs TTX # *p* < 0.05) and the relative BAX/BCL-2 (i) (*n* = 5, Kruskal–Wallis test, *p < 0.05, Dunn’s multiple comparisons test: control vs Gbz *p* > 0.05, control vs TTX *p* > 0.05, Gbz vs TTX # *p* < 0.05)

To investigate whether *Bax* and *BCL-2* expression is regulated by neuronal activity, we next performed in vitro experiments with cultured cortical neurons. In primary cultures, the occurrence of cell death is shown to be affected by the gestational age at which the cortical neurons are dissociated [34], but also by the expression of activity [25] and thus the culturing period, which is necessary for these neurons to form functional connections and to display spontaneous activity. Primary cortical neurons generated from newborn (P0) pups first displayed spontaneous activity at DIV7 [49], and a significant, activity-dependent loss of neurons was only observed after 9 days in vitro [25, 50]. We thus pharmacologically modulated spontaneous activity levels in primary cortical cultures generated from P0 mice from DIV10 onwards. Subsequently, we examined expression levels of *Bax* and its pro-survival counterpart *BCL-2* compared to untreated control conditions by multiplexed FISH and Western blot analysis. To increase neuronal activity, we applied the GABA_A_-receptor antagonist gabazine (Gbz, 10 µM). To block neuronal spiking activity, we applied the sodium channel blocker tetrodotoxin (TTX, 1 µM) at DIV10. Multiplexed FISH against the IEG *cFos* (positive control) as well as against *Bax* and *BCL-2* was performed after 6 h of activity modulation to investigate the acute effect of this manipulation (Fig. 3a). As expected, Gbz application induced an increase in neuronal activity observable as an increase in mean *cFos* signal intensity per cell (Fig. 3b, control: 288.5 ± 29.96 vs Gbz: 885.1 ± 140.2 vs TTX: 258.3 ± 14.21 a.u., one-way ANOVA, *p* < 0.0001). *Bax* expression in neurons, analyzed as a number of *Bax* puncta per cell, was significantly higher in the absence of activity (TTX) compared with control and increased neuronal activity conditions (Gbz) (Fig. 3c, control: 12.25 ± 1.12 vs Gbz: 10.03 ± 0.82 vs TTX: 24.59 ± 1.36 puncta/cell, one-way ANOVA, *p* < 0.0001). In contrast, disinhibition of network activity resulted in a non-significant change in *Bax* mRNA expression compared with untreated control cultures. Neither modification of activity had a significant effect on *BCL-2* mRNA expression (Fig. 3d, control: 1.56 ± 0.27 vs Gbz: 1.72 ± 0.28 vs TTX: 1.95 ± 0.33 puncta/cell, one-way ANOVA, *p* > 0.05). As a result, also the individual *Bax/BCL-2* ratio, i.e., the relative number of *Bax* and *BCL-2* puncta within individual cells, was significantly increased by blockade of neuronal activity (Fig. 3e, control: 6.45 ± 0.78 vs Gbz: 4.95 ± 0.53 vs TTX: 13.16 ± 1.50, one-way ANOVA, *p* < 0.0001). Since the proportions of *Bax*-only cells were similar in all experimental conditions, *BCL-2*-negative cells were not considered for ratio calculation (control: 32/71, Gbz: 30/74, TTX: 32/75 cells). For investigation of the long-lasting effects of the activity manipulations, we performed Western blot analysis of BAX and BCL-2 on DIV13 after 72 h of pharmacological treatment (Fig. 3f). In contrast to the acute effect on the mRNA level, we did not see significant changes in BAX protein expression (Fig. 3g, control: 1.00 ± 0.16 vs Gbz: 0.78 ± 0.15 vs TTX: 0.82 ± 0.05, one-way ANOVA, *p* > 0.05). However, when neuronal activity was increased, BCL-2 protein levels were significantly higher compared to TTX-treated cultures (Fig. 3h, control: 1.00 ± 0.10 vs Gbz: 1.40 ± 0.14 vs TTX: 0.75 ± 0.17, one-way ANOVA, *p* < 0.05). As a result, the resulting BAX/BCL-2 ratio was also significantly reduced in Gbz-treated cultures compared to TTX-treated cultures (Fig. 3i, control: 1.00 ± 0.11 vs Gbz: 0.57 ± 0.09 vs TTX: 1.51 ± 0.47, Kruskal–Wallis test, *p* < 0.05).

In conclusion, acute blockade of neuronal activity led to an increase in *Bax* expression on the mRNA level, while, on the protein level, long-lasting disinhibition of network activity resulted in increased BCL-2. Hence, manipulation of electrical activity resulted in a significant regulation of the intrinsic apoptotic pathway, such that in both ways higher activity was associated with lower relative BAX/BCL-2 expression.

### Active neurons show lower *Bax* and higher *BCL-2* mRNA expression and are more likely to survive

**Fig. 4:**
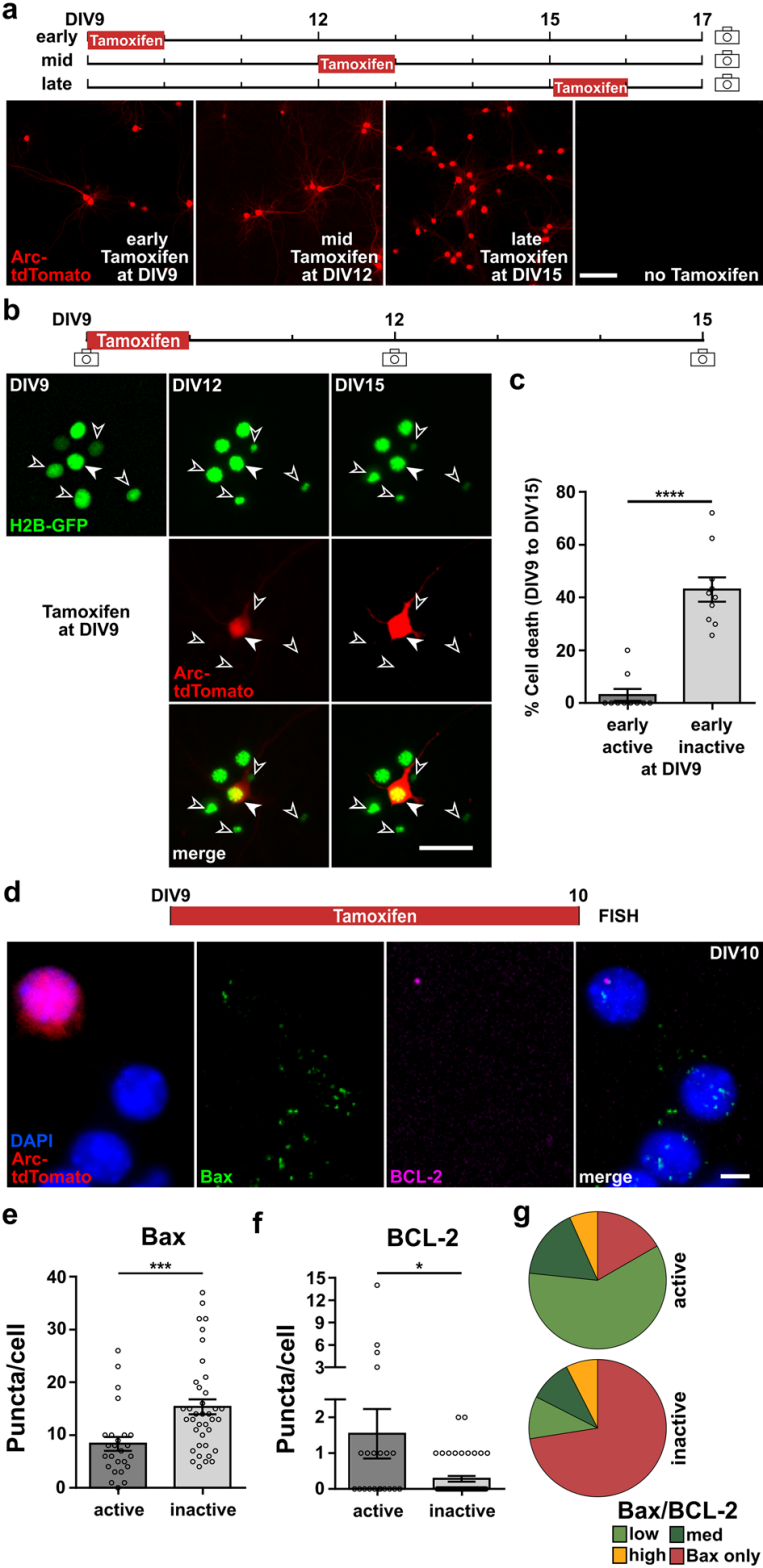
Pro-apoptotic *Bax* is downregulated and the pro-survival *BCL-2* is upregulated in active neurons. **a** *Arc*-dependent tdTomato expression at DIV17 in primary cortical neurons from newborn (P0) transgenic mice upon one-time application of tamoxifen at different developmental stages in vitro (scale bar = 100 µm). **b** Representative longitudinal image sequence of neurons from DIV9 to DIV15. One-time tamoxifen application at DIV9 induces permanent Arc-tdTomato labeling of early active neurons (filled arrow). The simultaneous expression of the nuclear tag H2B-GFP allows the unbiased identification of apoptotic neurons (unfilled arrows) with nuclear condensation (scale bar = 50 µm). **c** Early active neurons have a higher chance of survival than inactive neurons (*n* = 10 fields of view from *N* = 3 cultures, paired Student’s *t* test, ****p < 0.0001). **d** Representative images of multiplexed FISH against *Bax* and *BCL-2* in Arc-Cre-tdTomato-positive (active) and Arc-Cre-tdTomato-negative (inactive) neurons at DIV10 after one-time tamoxifen application 24 h earlier (scale bar = 5 µm). **e** *Bax* expression is significantly lower in early active neurons compared to inactive neurons (*n* = 28, 41 neurons from *N* = 3 culture preparations, Mann–Whitney test, ***p < 0.001). **f** Early active neurons show higher *BCL-2* expression than inactive neurons. (*n* = 22, 46 neurons from *N* = 3 culture preparations, Mann–Whitney test, *p < 0.05). **g** Group-wise comparison of *Bax/BCL-2* ratio. *Bax/BCL-2* ratio was grouped as follows: low = relative ratio 1–10, medium (med) = relative ratio 11–30, high = relative ratio > 30, *Bax* only = no detectable *BCL-2* expression (Chi-square, *p* < 0.0001)

During early phases of development, neuronal activity in primary cortical networks is limited to a few spontaneously active neurons and only later evolves into synchronous activity patterns [49, 51]. This early pattern of spontaneous activity in single neurons has recently been shown to be predictive of their later survival [25]. Yet, it remains unclear how the activity state of single neurons instructs cell death versus survival decisions. To assess whether single neuron activity affects the expression of the pro-apoptotic factor BAX, the anti-apoptotic BCL-2, and subsequently the BAX/BCL-2 pathway and whether an activity-dependent regulation of this pathway may lead to subsequent cell death or survival, we used primary cortical cultures from newborn (P0) Arc^CreERT/+^/R26^AI14/+^ mice [40, 41]. In these transgenic cultures, neurons express tdTomato under the activity-regulated cytoskeleton-associated protein (Arc) promoter in a tamoxifen-dependent manner. Like *cFos*, *Arc* belongs to the group of IEGs known for strong upregulation upon neuronal activity. In agreement with a developmental increase in the proportion of active neurons [25, 51], the proportion of tdTomato-positive neurons was strongly increased when tamoxifen was applied at later stages (DIV12 and DIV15) as compared to earlier time points (DIV9) (Fig. 4a). Upon addition of tamoxifen to mature neuronal cultures at DIV15, almost all neurons were tdTomato positive and thus considered spontaneously active. Sparse labeling of a few active neurons through tamoxifen application at DIV9 thus allows the analysis of expression profiles of early active versus inactive neurons. To compare survival rates of single tdTomato-positive and -negative neurons, we first followed their respective cell fate until DIV15. In this subset of experiments, cultures were transduced with a histone-fused nuclear GFP (H2B-GFP, Fig. 4b), which allowed the unbiased identification of apoptotic neurons based on longitudinal changes in chromatin compaction and nuclear size [27, 38]. In agreement with previous data [25], the percentage of cell death of tdTomato-positive, active neurons at DIV9 was 14 times lower compared to tdTomato-negative, inactive neurons (Fig. 4c, active: 3.11 ± 2.18 vs inactive: 43.08 ± 4.58% of cell death, paired *t*-test, *p* < 0.0001). Thus, this model allows us to compare early active neurons with a prospective cell survival fate with the profile of early inactive neurons with a higher likelihood of an apoptotic cell fate.

Therefore, we performed multiplexed FISH against *Bax* and *BCL-2* on cultures treated with 4-OH-Tam at DIV9 to compare expression levels in early active versus inactive neurons at DIV10 (Fig. 4d). In line with the results upon acute modulation of activity, active neurons showed significantly lower mRNA expression of *Bax* as compared to inactive neurons (Fig. 4e, active: 8.36 ± 1.32 vs inactive: 15.34 ± 1.44 puncta/cell, Mann–Whitney test, *p* < 0.001). The overall expression level of *BCL-2* was very low, but *BCL-2* expression was almost exclusively observed in tdTomato-positive, i.e., active neurons (Fig. 4f, active: 1.55 ± 0.69 vs inactive: 0.28 ± 0.08 puncta/cell, Mann–Whitney test, *p* < 0.05). The absence of *BCL-2* signals in a considerable subset of neurons (34/70 neurons) precluded the calculation of individual *Bax/BCL-2* ratios and its comparison between active and inactive neurons. Yet, to assess the relative profiles, we performed a group-wise analysis of neurons: low *Bax/BCL-2* ratio (1–10 puncta/cell), medium (11–30 puncta/cell), high (> 30 puncta/cell), and *Bax* only = *BCL-2* expression not detectable (Fig. 4g). The distribution of groups was significantly different between active and inactive neurons (Chi-square, *p* < 0.0001). In the active neuron population, most neurons showed a low *Bax/BCL-2* ratio (18/30 neurons) and only a few were *BCL-2*-negative (5/30 neurons). In contrast, most inactive neurons were devoid of *BCL-2* expression (*Bax* only 29/40 neurons) and otherwise equally distributed within the other groups (low = 4/40 neurons, medium = 4/40 neurons, high = 3/40 neurons).

In summary, expression of the pro-apoptotic factor *Bax* was significantly lower in spontaneously active neurons compared with inactive neighboring neurons, while its pro-survival counterpart *BCL-2* was upregulated. This suggests that the activity-dependent downregulation of the *Bax/BCL-2* ratio contributes to significantly higher survival rates in neurons active at the early stages of network development.

### Increased electrical activity prevents cell death in a pro-apoptotic environment

**Fig. 5:**
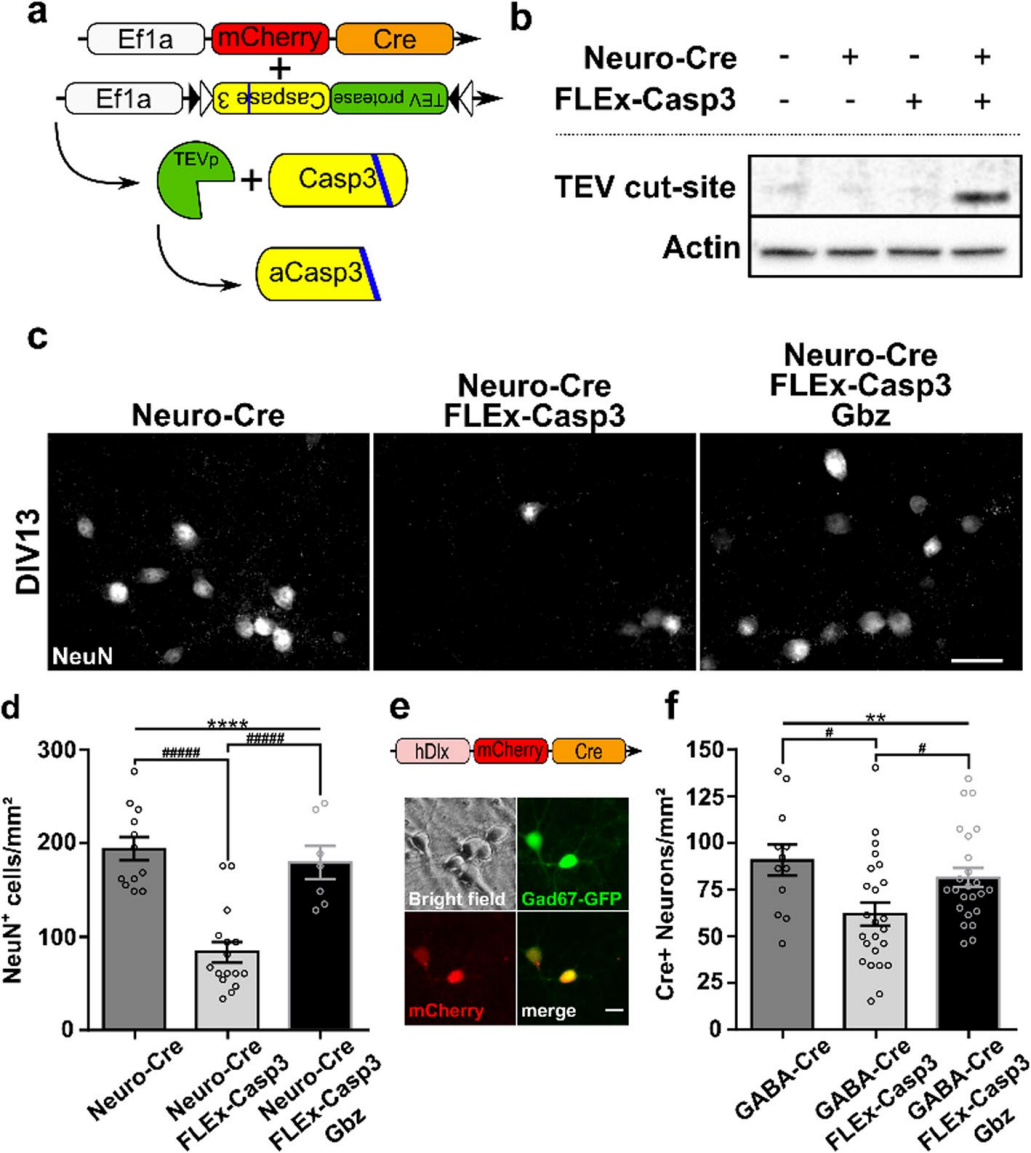
Increased electrical activity prevents cell death even in a pro-apoptotic environment. **a** Schematic overview of dual AAV approach for aCASP3 overexpression. **b** Western blots against the TEV cut-site on HEK293T cell lysates transfected with neither, one of the two, or both constructs. **c** Representative NeuN immunostainings of cortical cultures generated from newborn (P0) mice at DIV13 transduced with Neuro-Cre only, co-transduced with Neuro-Cre and FLEx-Casp3, or co-transduced with Neuro-Cre and FLEx-Casp3 and treated with Gbz at DIV10 (scale bar = 20 µm). **d** Overexpression of aCASP3 leads to cell death and thus a significantly reduced density of NeuN-positive cells. Disinhibition of network activity prevents this aCASP3-dependent loss of NeuN-positive cells (*n* = 12, 16, 7 fields of view from *N* = 3 cultures each, one-way ANOVA, ****p < 0.0001, post hoc Tukey’s multiple comparisons test: Neuro-Cre vs Neuro-Cre + FLEx-Casp3, ^####^*p* < 0.0001, Neuro-Cre vs Neuro-Cre + FLEx-Casp3 + Gbz, ns, Neuro-Cre + FLEx-Casp3 vs Neuro-Cre + FLEx-Casp3 + Gbz, ^####^*p* < 0.0001). **e** Specific targeting of interneurons through co-transduction with an hDlx-driven Cre construct was confirmed by colocalization of mCherry-Cre with GAD67-GFP (scale bar = 10 µm). **f** Increasing neuronal network activity has a neuroprotective effect in aCASP3-overexpressing GABAergic interneurons (*n* = 24 fields of view from *N* = 4 cultures each, one-way ANOVA, **p < 0.01, Tukey’s multiple comparison test: GABA-Cre vs GABA-Cre + FLEx-Casp3, #*p* < 0.05, GABA-Cre vs GABA-Cre + FLEx-Casp3 + Gbz, *p* > 0.05, GABA-Cre + FLEx-Casp3 vs GABA-Cre + FLEx-Casp3 + Gbz, #*p* < 0.05)

Cortical caspase activity is highest at very early developmental time points, yet neuronal apoptosis peaks only around the end of the first postnatal week in the murine cortex (Fig. 1, [16]). In agreement with recent studies [52], this suggests that although CASP3 is an effector of apoptosis [53], developing neurons can tolerate relatively high levels of caspase activity without becoming apoptotic, but also that activity-dependent modulation of apoptosis likely acts via different signaling pathways. To test whether neuronal activity can critically affect neuronal CASP3 tolerance and hence survival rates, we used a dual AAV approach to overexpress active CASP3 (aCASP3) in a Cre-dependent manner. The first construct encoded the Cre-recombinase with the reporter protein mCherry and the second was a double-floxed, inversely oriented construct that carried the sequence for caspase 3 and the tobacco etch virus protease (TEVp). The caspase recognition site is replaced by the TEV cut-site (FLEx-Casp3) [54], and thus, this model resembles the natural cleavage-dependent activation of CASP3 (Fig. 5a). In the first series of experiments, both constructs were controlled by the pan-neuronal promoter elongation factor 1a (Ef1a, short: Neuro-Cre, Suppl. Figure 4a, b). To test for the efficacy and reliability of this double transgenic approach, we first transfected HEK293T cells with either one of the two constructs, or both constructs, respectively. The results confirmed that the TEV cut-site was detectable only if all genetic elements were expressed (double transfected cells) and that the overexpressed CASP3 is activated only through the Cre-dependent expression of TEVp and the genetically modified CASP3 (Fig. 5b).

Next, primary cortical neurons were transduced with Neuro-Cre at DIV1-3 to ensure high levels of Cre-Recombinase/mCherry expression. Cultured neurons were first allowed to grow into networks without overexpression of aCASP3 and only later subjected to aCASP3-dependent cell death by the delayed addition of FLEx-Casp3-AAV at DIV7 (Suppl. Figure 4c, d). As expected, neurons overexpressing aCASP3 had higher rates of cell death that led to a significant reduction of cell numbers at DIV13 compared to control cultures only transduced with Neuro-Cre (Fig. 5c, d). Interestingly, this apoptotic loss of neurons was prevented when neuronal network activity was increased by the application of Gbz at DIV10 (Neuro-Cre: 194.1 ± 12.44 vs Neuro-Cre + FLEx-Casp3: 83.53 ± 10.86, vs Neuro-Cre + FLEx-Casp3 + Gbz: 179.4 ± 17.54 cells/mm^2^, one-way ANOVA, *p* < 0.0001). Before activity modulation, no significant reduction in neuronal numbers was detectable in aCASP3-overexpressing cultures (Suppl. Figure 4e). This prevention of cell death was not simply due to enhanced survival of non-transduced neurons, as it was also significant for aCASP3-overexpressing cells (Suppl. Figure 4f). In line with this, aCASP3-overexpressing cells were fully functional and able to participate in network activity (Suppl. Figure 5).

To address the question whether this activity-dependent prevention of cell death under sustained CASP3 expression is a cell-type-specific effect, we targeted the aCASP3 overexpression specifically to GABAergic interneurons by combining the FLEx-Casp3 construct with Cre-recombinase under the interneuron specific hDlx promotor (GABA-Cre) (Fig. 5e). Specificity of this approach was confirmed in primary cortical cultures from GAD67-GFP mice (Suppl. Figure 6). Following the same experimental regime and analyzing the density of Cre-recombinase-positive interneurons showed that aCASP3-overexpressing interneurons died more frequently than interneurons transduced only with the GABA-Cre construct. Again, cell death could be prevented by increasing neuronal network activity through disinhibition starting at DIV10 (Fig. 5f, GABA-Cre: 90.72 ± 8.21 vs GABA-Cre + FLEx-Casp3: 61.92 ± 6.11 vs GABA-Cre + FLEx-Casp3 + Gbz: 81.44 ± 5.04 cells/mm^2^, one-way ANOVA, *p* < 0.01).

Taken together, increased electrical activity prevented the apoptotic neuronal loss in both excitatory and inhibitory cortical neurons under prevalent aCASP3 overexpression, i.e., a strong pro-apoptotic background. This pro-survival effect of electrical activity is occurring in different neuronal populations.

### Activity-dependent downregulation of *Bax*/*BCL-2* ratio in pro-apoptotic conditions

The neuroprotective effect of activity under prevalent CASP3 activation could either be explained by direct inhibition of the CASP3 activity, or an increased tolerance via a CASP3-independent pathway.

**Fig. 6:**
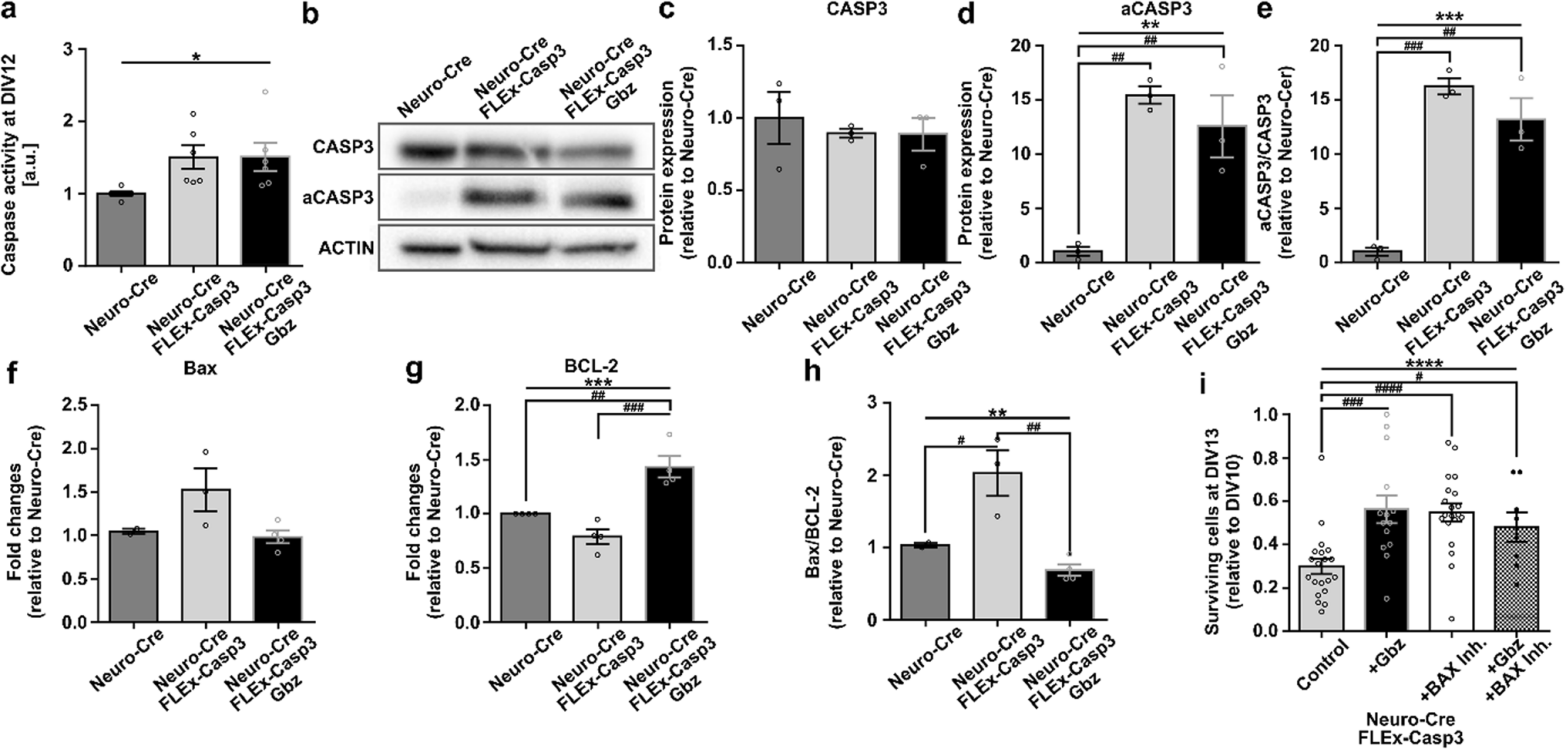
Activity-dependent downregulation of the BAX/BCL-2 pathway increases neuronal tolerance to aCASP3 expression. **a** Caspase 3/7 activity measured by luminescent Caspase-Glo assay is significantly increased by aCASP3 overexpression in cortical neurons from newborn (P0) mice at DIV12 regardless of Gbz treatment (*n* = 6 measurements from *N* = 6 cultures, one-way ANOVA: * *p* < 0.05). **b** Representative Western blots against full-length and cleaved CASP3 in non-treated neuronal cultures transduced with only Neuro-Cre or Neuro-Cre + FLEx-Casp3 and Gbz-treated cultures transduced with Neuro-Cre + FLEx-Casp3. **c**, **d** Quantification of Western blots against full-length CASP3 (**c**) and aCASP3 (**d**) in single-transduced Neuro-Cre cultures and double-transduced Neuro-Cre + FLEx-Casp3 non-treated and Gbz-treated cultures (*n* = 3, one-way ANOVA, *p* > 0.05, **p < 0.01, post hoc Tukey’s multiple comparisons test: Neuro-Cre vs Neuro-Cre + FLEx ^##^*p* < 0.01, Neuro-Cre vs Neuro-Cre + FLEx-Casp3 + Gbz ^##^*p* < 0.01, Neuro-Cre + FLEx-Casp3 vs Neuro-Cre + FLEx-Casp3 + Gbz *p* > 0.05). **e** The aCASP3/CASP3 ratio in aCASP3-overexpressing cultures is significantly higher regardless of the treatment compared to single-transduced Neuro-Cre cultures (*n* = 3, one-way ANOVA ***p < 0.001, Tukey’s multiple comparisons test: Neuro-Cre vs Neuro-Cre + FLEx-Casp3 ^###^*p* < 0.001, Neuro-Cre vs Neuro-Cre + FLEx-Casp3 + Gbz ^##^*p* < 0.01, Neuro-Cre + FLEx-Casp3 vs Neuro-Cre + FLEx-Casp3 + Gbz *p* > 0.05). **f**, **g** Quantitative real-time PCR for *Bax* (**f**) (*n* = 2; 3; 4 measurements from *N* = 2; 3; 4 cultures, one-way ANOVA *p* > 0.05) and *BCL-2* (**g**). *BCL-2* is significantly upregulated when aCASP3-overexpressing cultures were treated with Gbz (*n* = 4 measurements from *N* = 4 cultures, one-way ANOVA ***p < 0.001, post hoc Tukey’s multiple comparisons test: Neuro-Cre vs Neuro-Cre + FLEx *p* > 0.05, Neuro-Cre vs Neuro-Cre + FLEx-Casp3 + Gbz ^##^*p* < 0.01, Neuro-Cre + FLEx-Casp3 vs Neuro-Cre + FLEx-Casp3 + Gbz.^###^*p* < 0.001). **h** Activity-dependent regulation of *Bax* and *BCL-2* expression leads to a significant change in the *Bax/BCL-2* ratio (*n* = 2; 3; 4 measurements from *N* = 2; 3; 4 cultures, one-way ANOVA **p < 0.01, post hoc Tukey’s multiple comparisons test: Neuro-Cre vs Neuro-Cre + FLEx-Casp3 ^#^*p* < 0.05, Neuro-Cre vs Neuro-Cre + FLEx-Casp3 + Gbz *p* > 0.05, Neuro-Cre + FLEx-Casp3 vs Neuro-Cre + FLEx-Casp3 + Gbz ^##^*p* < 0.01). **i** The fraction of surviving cells at DIV13 in double-transduced cultures was similarly increased by the application of Gbz or the BAX inhibitor peptide V5. No additive effect was present when pro-apoptotic cultures were treated with both Gbz and BAX inhibitor peptide V5 (*N* = 4, *n* = 19, 14, 20, 8, one-way ANOVA ****p < 0.0001, post hoc Tukey’s test: Neuro-Cre + FLEx-Casp3 vs Neuro-Cre + FLEx-Casp3 + Gbz ^###^*p* < 0.001, Neuro-Cre + FLEx-Casp3 vs Neuro-Cre + FLEx-Casp3 + Bax Inh. ^####^*p* < 0.001, Neuro-Cre + FLEx-Casp3 vs Neuro-Cre + FLEx-Casp3 + Gbz + Bax Inh. # *p* < 0.05 Neuro-Cre + Flex-Casp3 + Gbz vs Neuro-Cre + FLEx-Casp3 + Bax Inh. *p* > 0.05, Neuro-Cre + Flex-Casp3 + Gbz vs Neuro-Cre + FLEx-Casp3 + Gbz + Bax Inh. *p* > 0.05, Neuro-Cre + FLEx-Casp3 + Bax Inh. vs Neuro-Cre + FLEx-Casp3 + Gbz + Bax Inh. *p* > 0.05)

To investigate whether electrical activity directly affects executor caspases, we measured at DIV12 caspase 3/7 activity by the luminescent Caspase-Glo assay in lysates from neuronal cultures with and without aCASP3 overexpression under control conditions as well as conditions of increased spontaneous activity (Gbz). As expected, caspase activity was significantly increased in aCASP3-overexpressing cultures compared to Neuro-Cre-only control cultures. Interestingly, positive modulation of electrical activity via Gbz application did not change the overall caspase 3/7 activity (Fig. 6a, Neuro-Cre: 1.00 ± 0.03 vs Neuro-Cre + FLEx-Casp3: 1.50 ± 0.16 vs Neuro-Cre + FLEx-Casp3 + Gbz: 1.51 ± 0.20, one-way ANOVA, *p* < 0.05). We further performed Western blots against CASP3 and aCASP3 (Fig. 6b). We did not detect any changes in the expression of the full-length protein between the groups (Fig. 6c, Neuro-Cre: 1.00 ± 0.18 vs Neuro-Cre + FLEx-Casp3: 0.89 ± 0.03 vs Neuro-Cre + FLEx-Casp3 + Gbz: 0.88 ± 0.11, one-way ANOVA, *p* > 0.05), but aCASP3 levels were prominently upregulated in cultures transduced with both constructs (Fig. 6d, Neuro-Cre: 1.00 ± 0.42 vs Neuro-Cre + FLEx-Casp3: 14.07 ± 0.78 vs Neuro-Cre + FLEx-Casp3 + Gbz: 12.59 ± 2.86, one-way ANOVA, *p* < 0.01). This increase resulted in a significantly changed aCASP3/CASP3 ratio (Fig. 6e, Neuro-Cre: 1.00 ± 0.39 vs Neuro-Cre + FLEx-Casp3: 16.24 ± 0.75 vs Neuro-Cre + FLEx-Casp3 + Gbz: 13.18 ± 1.95, one-way ANOVA, *p* < 0.001). Because disinhibition of network activity did not alter the enzymatic activity or protein expression, we can rule out the possibility that the neuroprotective effect of increased activity directly affected CASP3 activity.

Next, we addressed whether the pro-survival effect of neuronal activity in aCASP3-overexpressing neurons could be caused by a regulation of the *Bax/BCL-2* pathway. First, we applied different pharmacological blockers of BCL-2 (ABT-199, HA14-1, obatoclax) to naïve cultures at DIV10. In line with the neuroprotective effect of overexpression of BCL-2 [55–57], specific blockade of BCL-2 led to neuronal cell death in a dose-dependent manner (Suppl. Figure 7). Next, we performed real-time PCR-based expression analysis of *Bax* and *BCL-2* in the same experimental groups as before (Neuro-Cre vs Neuro-Cre + FLEx-Casp3 + Gbz). Under untreated conditions, *Bax* mRNA expression levels were slightly but not significantly elevated in Neuro-Cre + FLEx-Casp3 cultures compared to Neuro-Cre control cultures. Meanwhile, upon conditions of elevated neuronal activity (Neuro-Cre + FLEx-Casp3 + Gbz), *Bax* expression levels were comparable to control conditions (Fig. 6f, Neuro-Cre: 1.05 ± 0.03 vs. Neuro-Cre+FLEx-Casp3: 1.53 ± 0.25 vs. Neuro-Cre + FLEx-Casp3 + Gbz: 0.99 ± 0.08, one-way ANOVA, *p* > 0.05). In contrast to *Bax*, expression of *BCL-2* was regulated in the way that overexpression of aCASP3 resulted in lower mRNA levels of *BCL-2*, whereas disinhibition of the network with Gbz led to a significant increase in BCL-2 expression even compared to control conditions (Fig. 6g, Neuro-Cre: 1.00 ± 0.00 vs Neuro-Cre + FLEx-Casp3: 0.79 ± 0.07 vs Neuro-Cre + FLEx-Casp3 + Gbz: 1.44 ± 0.10, one-way ANOVA, *p* < 0.001).

Taken together, the mild regulation of *Bax* and the strong regulation of *BCL-2* resulted in a strong shift in the *Bax/BCL-2* ratio. Untreated cultures that overexpress aCASP3 showed double the *Bax/BCL-2* ratio compared to controls, indicating that they were more prone to apoptosis. Interestingly, this difference was even greater when compared to aCASP3-overexpressing cultures with increased activity levels (Fig. 6h, Neuro-Cre: 1.04 ± 0.03 vs Neuro-Cre + FLEx-Casp3: 2.03 ± 0.32 vs Neuro-Cre + FLEx-Casp3 + Gbz: 0.69 ± 0.08, one-way ANOVA, *p* < 0.01).

To further investigate the mechanisms involved in the anti-apoptotic effect of electrical activity, we blocked BAX by application of the BAX inhibitor peptide V5 (10 µM [58]). Here, we added either Gbz, the BAX inhibitor, or both to aCASP3-overexpressing cultures at DIV10 and compared the percentage of surviving cells at DIV13 to non-treated aCASP3-overexpressing cultures. Cultures in which BAX dimerization was blocked by peptide V5 application showed a similar cell survival rate to those with elevated activity levels. In line with the hypothesis that the neuroprotective effect of increased activity is a BAX-dependent regulation, there was no additive effect, if neurons were treated with both, Gbz and the BAX inhibitor (Fig. 6i, Neuro-Cre + FLEx-Casp3: 30.03 ± 3.52 vs Neuro-Cre + FLEx-Casp3 + Gbz: 56.40 ± 6.41 vs Neuro-Cre + FLEx-Casp3 + BAX Inhibitor: 54.84 ± 4.08%, one-way ANOVA, *p* < 0.0001).

In summary, our results suggest that the anti-apoptotic effect of electrical activity could not be explained by direct regulation of the caspase 3/7 activity, but rather by a downregulation of the pro-apoptotic BAX/BCL-2 ratio. In opposite to the neurotoxic effect of the pharmacological blockade of BCL-2 [59, 60], an increase in electrical activity has a similar and non-additive neuroprotective effect in a pro-apoptotic environment as a pharmacological blockade of BAX activity.

## Discussion

The homeostatic removal of cortical neurons during early brain development requires complex regulation by multiple control mechanisms partly working in parallel. Our study investigated whether the BAX/BCL-2 pathway serves as a regulatory mechanism and if electrical activity may be its potential set point during cortical development. The main observations of the present study are as follows: (I) aCASP3 activity is highest in the cortex of newborn mice and then sharply declines, while developmental cell death peaks at the end of the first postnatal week; (II) during the first postnatal days, the upregulation of BAX expression in the cortex is accompanied by the downregulation of BCL-2, resulting in the highest BAX/BCL-2 ratio when neuronal death rates are high; (III) at the level of individual neurons, acute pharmacological blockade of network activity leads to an upregulation of *Bax* expression and thus to a shift in the BAX/BCL-2 ratio toward the pro-apoptotic side; (IV) activity-dependent regulation of *Bax/BCL-2* is also observed in spontaneously active neurons during early network development. Not only is the *Bax/BCL-2* ratio lower in active neurons but also a large proportion of inactive neurons lack *BCL-2* expression; (V) disinhibition of network activity prevents the death of excitatory and inhibitory cortical neurons overexpressing aCASP3; and (VI) the neuroprotective effect of activity is not the result of reduced caspase activity but is associated with a downregulation of the *Bax/BCL-2* ratio. Notably, increasing electrical activity has a similar and non-additive neuroprotective effect as the pharmacological blockade of BAX activity.

During postnatal murine development, death rates of cortical neurons as quantified by aCASP3-positive neurons [18, 19, 34], number of surviving neurons [19], or TUNEL-positive neurons [this study, 19, 20, 49] is highest at the end of the first postnatal week, despite regionally specific differences in the extent of this developmental cell death [18]. Activation of the executioner CASP3 is considered an important marker of apoptosis and apoptotic neurons display strong activation of CASP3 during the final phase of death [38]. Yet, our data show that total cortical caspase activity is significantly higher upon birth and then sharply declines, while the peak of cortical cell death rate occurs only later. The observed high caspase activity might be explained by non-apoptotic roles of aCASP3 [61, 62]. It has been shown that restricted and localized CASP3 activation is involved in different homeostatic mechanisms such as synaptic pruning [63], axon pathfinding [64], and axon arborization [65]. All of these mechanisms are especially important during early postnatal development and require a certain tolerance for caspase activity in immature neurons. But also, the decreasing caspase activity in the postnatal cortex suggests that caspase activity does not necessarily correlate with cell death of immature cortical neurons, thus ruling out direct regulation of caspase activity by neuromodulatory mechanisms such as electrical activity.

BAX and other members of the pro-apoptotic group of the BCL-2 family play critical or essential roles in developmental apoptosis [8–12, 66]. Our data, corroborated by previous literature [47, 67], show that developmental upregulation of BAX during the first postnatal days is accompanied by downregulation of BCL-2 protein expression, resulting in a BAX/BCL-2 ratio that is highest around P7 precisely when cortical cell death is high. While this developmental upregulation of the *BAX/BCL-2* ratio is also seen on the mRNA level, the absolute number of RNA particles encoding for *Bax* and *BCL-2* both increases with development, suggesting a more nuanced developmental regulation on the post-transcriptional level. Further, we cannot rule out the possibility that cell-type-specific developmental effects contribute to this regulation. If so, these effects most likely originate from neuronal subtypes due to the predominance of neurons in the early postnatal cortex [48]. Other anti-apoptotic members of the BCL-2 family, i.e., MCL-1 and BCL-xL, are also important for the proper development of the cortex but only play key roles at earlier time points [13, 15]. Furthermore, our data are in line with early findings demonstrating that BCL-2 overexpression protects neurons from developmental apoptosis [17]. During the developmental period examined in this study, cortical network activity matures from sparse, uncorrelated events to highly correlated bursting activity of strongly interconnected neurons[22, 68]. Primary cortical cultures also display network-wide bursting activity [49], and both in vitro and in vivo high-frequency oscillatory activity is especially critical for cell survival [18, 27, 69]. The neuroprotective effect is at least partly mediated through Ca^2+^ influx upon NMDA-receptor activation and voltage-dependent Ca^2+^ channels [24, 70, 71]. An increase in intracellular Ca^2+^ can lead to upregulations of different pro-survival pathways in neurons, i.e., CaMK pathway [72, 73], CREB [74, 75], and/or calcineurin [20]. At the same time, the activity-dependent regulation of neuronal cell death is *Bax* dependent [19, 20, 34, 76]. Data from our recent report suggest that bursting activity, which increases neuronal survival, is associated with lower *Bax/BCL-2* ratios in cortical neurons [27]. Consistent with this, pharmacological blockade of activity in primary cortical neurons leads to an upregulation of *Bax* and thus to a shift of the *Bax/BCL-2* toward the pro-apoptotic side. Disinhibition of the network activity leads to an upregulation of *cFos*, a classical IEG that is regulated by Ca^2+^ influx when neurons show bursting activity [27, 77] but only has a non-significant opposite effect on the *Bax/BCL-2* ratio. On the longer timescale, a consistent activity-dependent regulation of the BAX/BCL-2 ratio was also observed on the protein level. However, on the protein level the individual regulation of both factors (BAX and BCL-2) did not resemble their regulation on the RNA level upon pharmacological modification of activity levels. In future, longitudinal monitoring of neuronal activity as well as RNA and protein expression levels in single neurons would be necessary to fully resolve the mechanisms underlying the activity-dependent and developmental regulation of the BAX/BCL-2 ratio on the transcriptional, translational and eventually the post-translational level. Nonetheless, these results suggest that in developing cortical neurons expression of the *Bax/BCL-2 ratio* is regulated in an activity-dependent manner.

To address whether the spontaneous occurrence of activity modulates the BAX/BCL-2 ratio in immature cortical neurons, we then compared expression levels of early active neurons with high survival rates to inactive neurons. In line with the developmental changes in activity and the observed downregulation of the *Bax/BCL-2* ratio after P7, active neurons display lower *Bax* mRNA levels and are almost exclusively *BCL-2* positive. It remains to be investigated how neuronal activity levels are translated into lower *Bax/BCL-2* expression. However, since activity patterns associated with higher survival rates are characterized by strong Ca^2+^ increases [24, 27, 36, 69], one potential mechanism is the activation of long-lasting neuroprotective mechanisms facilitated through CREB activation [75]. In addition, other control mechanisms likely act in parallel to control cell death decisions [78]. For example, cell survival upon induction of bursting activity was blocked by antagonizing phosphatidylinositol-3 kinase (PI3K) action in vitro [35], and activity-dependent cell death of interneurons is regulated by PTEN expression, an antagonist of the PI3K/Akt pathway [19].

Consistent with the essential role of *Bax* in the execution of developmental apoptosis [8–13, 17, 66], spontaneously active neurons with higher survival chances show an activity-dependent downregulation of the *Bax/BCL-2* ratio. At the network level, the participation of neurons in network activity increases with their functional and structural maturation [25], concomitant with a decrease in the overall BAX/BCL-2 ratio as developmental cell death subsides. BCL-2 antagonizes pro-apoptotic BAX function [6]. At the same time, other cell death-related caspase-independent signaling pathways are also inhibited by BCL-2, contributing to its neuroprotective function in neurons [55, 79].

For the regulation of developmental cell death, two default settings are principally conceivable: (I) all neurons are set to survive and switch to apoptosis only when they do not exhibit and/or receive electrical activity at a certain developmental time point or (II) all neurons are prone to die and activity selectively rescues functional, connected neurons. The latter hypothesis is supported by the fact that neurons show a natural tolerance to caspase activity, as seen by the high caspase activity in neonatal mice at a time of relatively low cell death. We see this tolerance in aCASP3-overexpressing neurons, as increased cell death is only detected after more than three days. Tolerance can be further increased by increasing activity levels, which is seen in both excitatory and inhibitory neurons. aCASP3-overexpressing neurons, which survive until DIV13 under network disinhibition, are functional and participate in network-wide events. This suggests that the neuroprotective potential of neuronal activity is not a tool for fine-tuning neuronal populations but a key factor in their establishment. Changes in neuronal activity do not affect caspase activity in aCASP3-overexpressing cultures, so the neuroprotective effect is not mediated via direct regulation of caspase activity. Interestingly, the *Bax/BCL-2* pathway is regulated such that increased neuronal activity shifts the *Bax/BCL-2* ratio to comparable levels with control, that is, toward the pro-survival side. The functional implication of the BAX/BCL-2 pathway is further corroborated by the finding that the pharmacological blockade of BAX activity leads to similar survival rates as the increase in electrical activity but has no additive effect. This suggests an activity-dependent increase of the sublethal threshold for CASP3 activity [62, 63] by downregulation of the BAX/BCL-2 ratio and caspase-independent mechanisms [80].

In conclusion, this study highlights the regulatory importance of electrical activity for neuronal survival and suggests that the neuroprotective effect of neuronal activity is regulated through the BAX/BCL-2 pathway. Our data are among the first to suggest that activity-dependent regulation of the BAX/BCL-2 pathway mediates the selective survival of functionally connected, active neurons during early cortical development when overall caspase activity is still high. At the same time, this increased tolerance for caspase activity in active neurons potentially also redirects CASP3 activity to non-lethal functions that are critical for proper development in early cortical networks. Taken together, this study shows that the activity-dependent regulation of the BAX/BCL-2 pathway protects immature cortical neurons from apoptotic death and at the same time hints at additional, non-apoptotic functions of the caspase protein family during early maturation of neurons.

### Supplementary Information

Below is the link to the electronic supplementary material.

Supplementary file1 (DOCX 1764 KB)

## Data Availability

The data that support the findings of this study are available from the corresponding author upon reasonable request.
