# Supplementary material for: Activity-dependent regulation of the BAX/BCL-2 pathway protects cortical neurons from apoptotic death during early development
Source: Cell Mol Life Sci. 2023 Jun 3;80(6):175. doi: 10.1007/s00018-023-04824-6 (PMC10239391; doi:10.1007/s00018-023-04824-6)
Supplement: Supplementary file 1 — Supplementary file1 (DOCX 1764 KB) [file 18_2023_4824_MOESM1_ESM.docx]

**Supplementary figures**

**
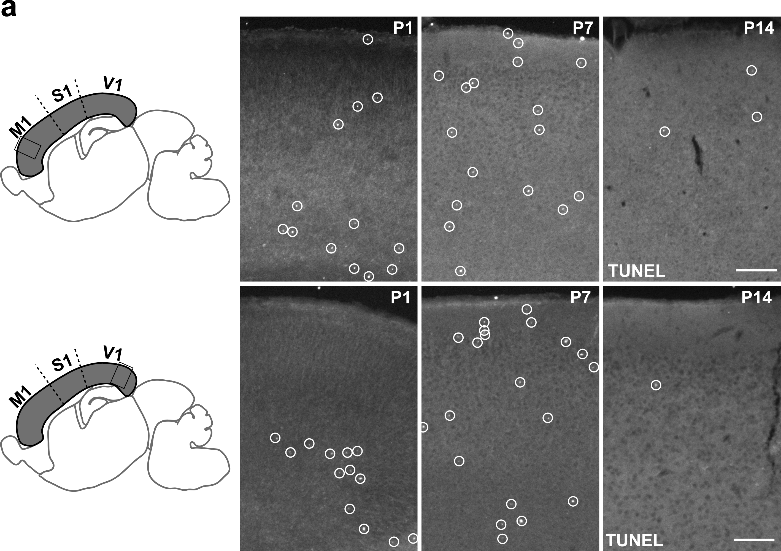
**

**Suppl. Fig. 1 | a)** Representative photographs of motor (M1, upper panel) and visual cortical region (V1, lower panel) of TUNEL stained sagittal cortical slices at P1, P7, and P14 (scale bar = 100 µm).

**
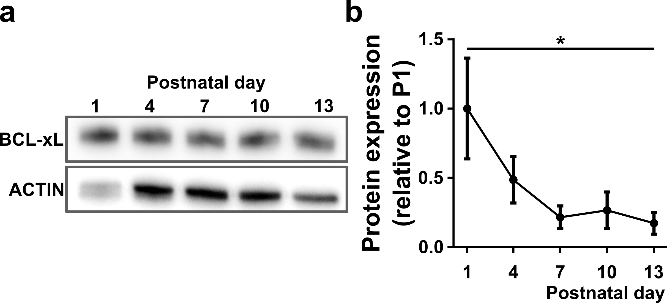
**

**Suppl. Fig. 2 | a)** Representative Western blot against BCL-xL at P1 to P13. **b)** Quantification of BCL-xL protein expression relative to P1 (n = 8 cortices from N = 8 mice, Kruskal-Wallis test, * p < 0.05).

**
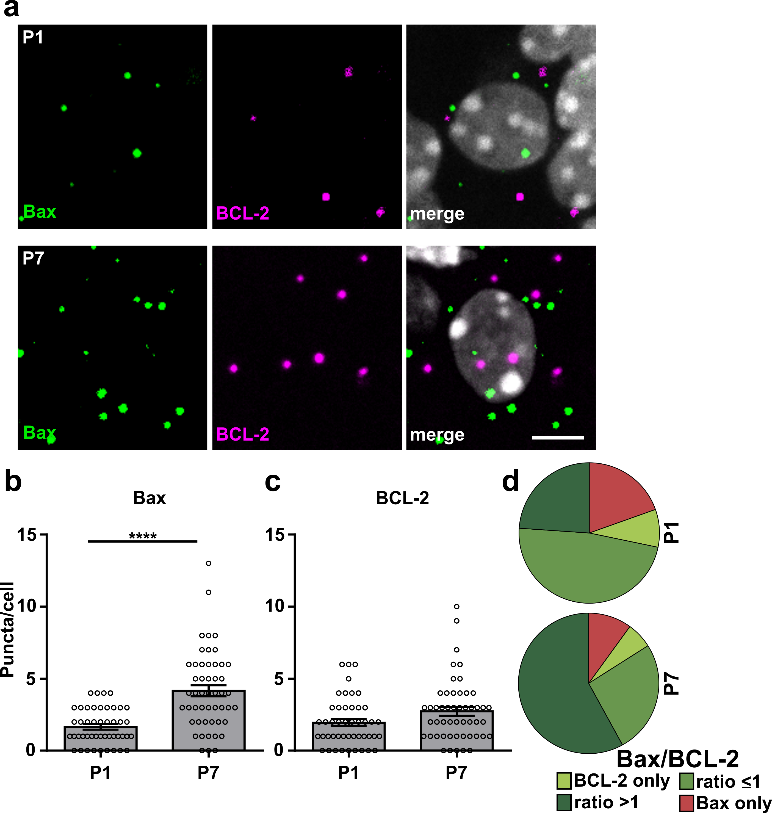
**

**Suppl. Fig. 3 | a)** Representative images of multiplexed FISH against *Bax* and *BCL-2* in single neurons of the cortical plate at P1 (upper panel), and layer II/III and IV at P7 (lower panel). **b-c)** Quantification of *Bax* (b) and *BCL-2* (c) puncta/cell (Student’s t test: Bax P1 vs. P7 **** p < 0.0001, BCL-2 P1 vs. P7 p > 0.05). **d)** Group-wise comparison of the individual *Bax/BCL-2* ratio, divided into 4 categories: “*BCL-2 only*” = no *Bax* puncta detected, “ratio ≤ 1” = individual *Bax/BCL-2* ratio ≤ 1, “ratio > 1” = individual *Bax/BCL-2* ratio > 1, “*Bax* only” = no *BCL-2* puncta detected (Chi-square ** p < 0.01).

**
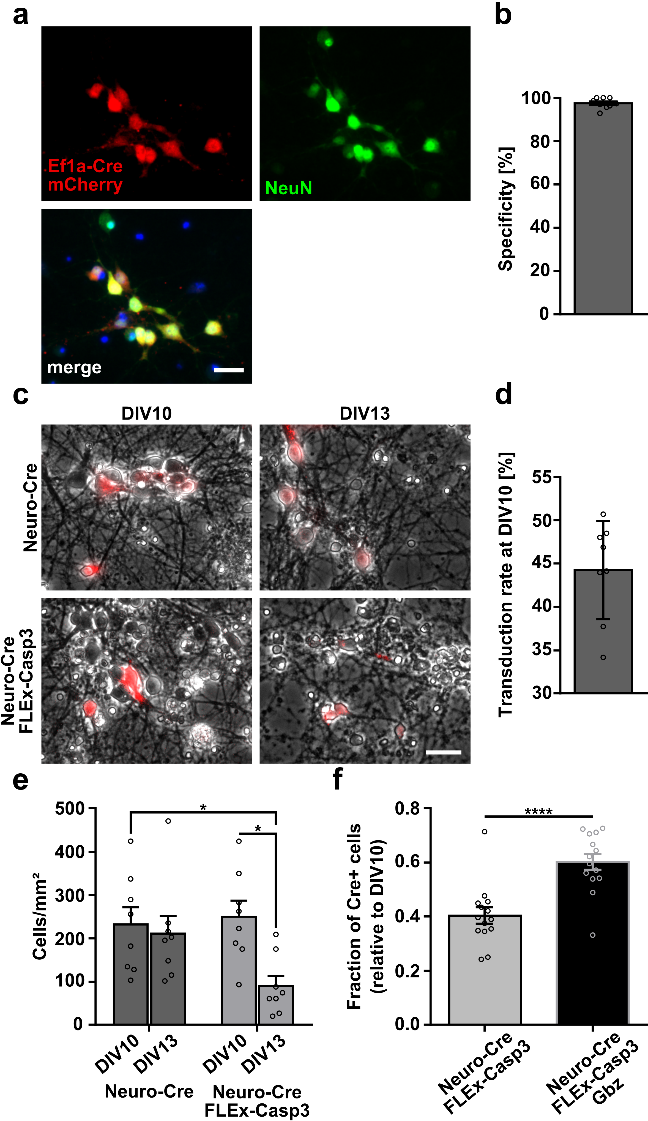
**

**Suppl. Fig. 4 | a)** Representative images of mCherry and NeuN staining from cultures, which were single-transduced with a high dose of the Neuro-Cre construct (scale bar = 20 µm). **b)** The specificity was calculated based on the colocalization of mCherrry and NeuN positive neurons (n = 9 fields of view from N = 3 independent culture preparations) **c)** Representative images of cultures transduced with Neuro-Cre or Neuro-Cre and FLEx-Casp3 at DIV10 and DIV13 (scale bar = 20 µm). **d)** Representative transduction rate calculated based on the relative proportion of Cre-positive vs Cre-negative cells in cultures transduced with Neuro-Cre or Neuro-Cre and FLEx-Casp3 at DIV10 (n = 8 fields of view from N = 4 independent culture preparations). **e)** Cell density in single and double transduced cultures at DIV10 and DIV13 quantified based on bright field images. Only at DIV13, significant differences are detectable (n = 8 fields of view from N = 4 independent culture preparations, Two-way ANOVA; Day in vitro * p < 0.05, Tukey’s multiple comparisons test: Neuro-Cre (DIV10) vs. Neuro-Cre (DIV13), p > 0.05, Neuro-Cre (DIV10) vs. Neuro-Cre+FLEx-Casp3 (DIV10), p > 0.05, Neuro-Cre (DIV10) vs. Neuro-Cre+FLEx-Casp3 (DIV13), p < 0.05, Neuro-Cre (DIV13) vs. Neuro-Cre+FLEx-Casp3 (DIV10), p > 0.05, Neuro-Cre (DIV13) vs. Neuro-Cre+FLEx-Casp3 (DIV13), p > 0.05, Neuro-Cre+FLEx-Casp3 (DIV10) vs. Neuro-Cre+FLEx-Casp3 (DIV13), p < 0.05). **f)** Increased electrical activity induced by application of Gbz from DIV10 on prevents cell death of Cre^+^ neurons that overexpress aCASP3 at DIV13 (n = 14 fields of view from 4 independent culture preparations, unpaired Student t-test, p < 0.0001).

**
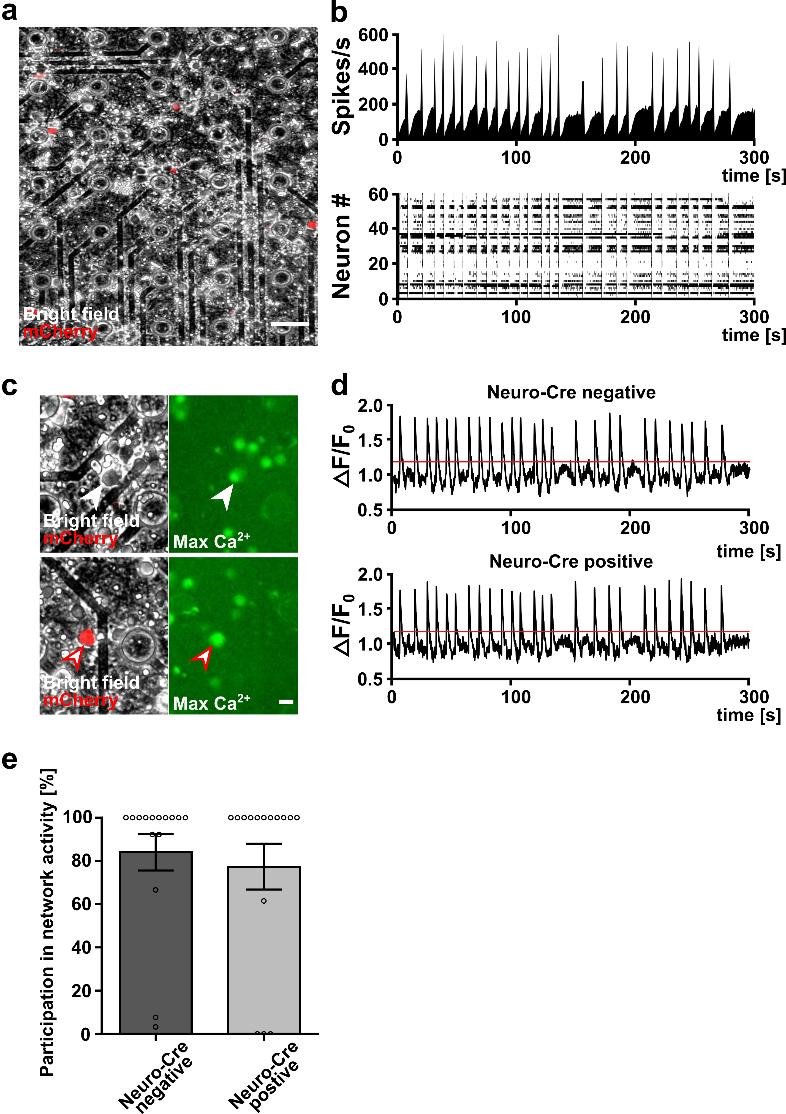
**

**Suppl. Fig. 5 | a)** Sparse transduction of neurons leads to only a few neurons overexpressing aCASP3 and thus, does not interfere with the normal maturation of the network (scale bar = 100 µm). **b)** Spike histogram and the associated raster plot of electrical network activity of culture at DIV13, treated with Gbz at DIV10. **c)** Representative merged images of a non-transduced (white arrow, upper, left panel) and aCASP3 overexpressing (red outlined arrow, lower, left panel) neuron from the neuronal culture shown in panel a and the maximum Ca^2+^ loading of the same neurons (right panels) (scale bar = 10 µm). **d)** Ca^2+^ traces of the neurons shown in panel c. aCASP3 overexpressing neurons are fully functionally integrated and follow the network activity. **e)** Percentage of network activity participation of Neuro-Cre negative and Neuro-Cre positive neurons (n = 15 cells from N = 3 independent preparations, unpaired Student’s t-test, p > 0.05).

**
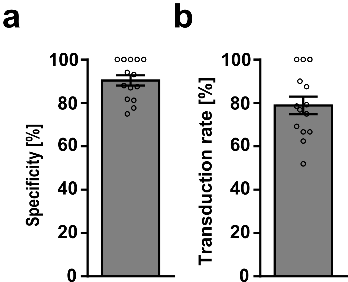
**

**Suppl. Fig. 6 | a)** Specificity of GABA-Cre construct. Percentage of colocalization between the GAD67-GFP and the mCherry signal. **b)** Transduction rate calculated based on the relative proportion of Cre-positive vs Cre-negative GAD67-GFP positive GABAergic interneurons (n = 14 fields of view from N = 3 independent culture preparations).


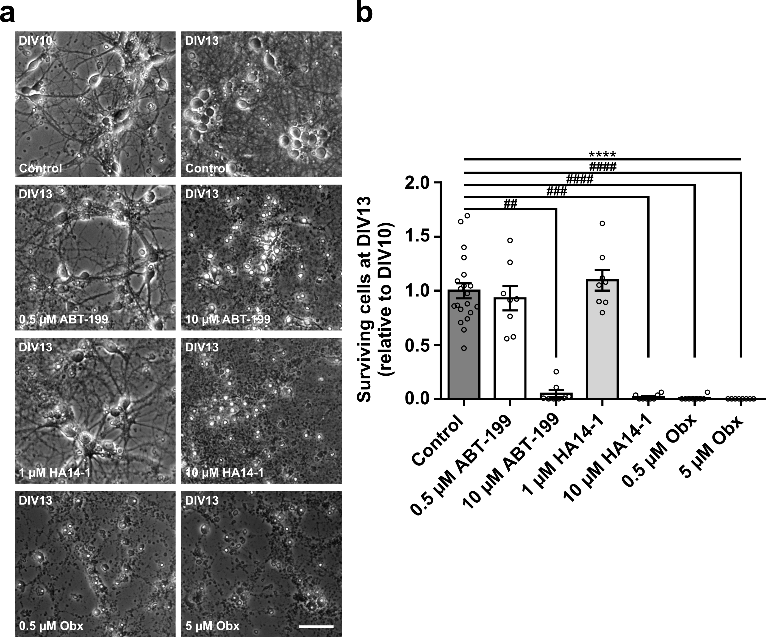


**Suppl. Fig. 7 |** **a)** Representative images of an untreated culture at DIV10 (upper left), and DIV13 (upper right), and cultures at DIV13 treated with different drugs and concentrations at DIV10. Cultures were treated with a low dose (left panel) or a high dose (right panel) of different BCL-2 specific inhibitors (ABT-199 = Venetoclax, HA14-1, or Obx = Obatoclax) (scale bar = 20 µm). **b)** Survival rate of neurons relative to DIV10 (n = 20, 8, 8, 8, 8, 8, 8 fields of view from 3 independent culture preparations, Kruskal-Wallis test, **** p < 0.0001, Dunn’s multiple comparisons test vs. Control: 0.5 µM ABT-199, p > 0.05, 10 µM ABT-199, p < 0.01, 1 µM HA14-1, p > 0.05, 10 µM HA14-1, p < 0.001, 0.5 µM Obx, p < 0.0001, 5 µM Obx, p < 0.0001).
